# Large scale international replication and meta-analysis study confirms association of the 15q14 locus with myopia. The CREAM consortium

**DOI:** 10.1007/s00439-012-1176-0

**Published:** 2012-06-05

**Authors:** Virginie J. M. Verhoeven, Pirro G. Hysi, Seang-Mei Saw, Veronique Vitart, Alireza Mirshahi, Jeremy A. Guggenheim, Mary Frances Cotch, Kenji Yamashiro, Paul N. Baird, David A. Mackey, Robert Wojciechowski, M. Kamran Ikram, Alex W. Hewitt, Priya Duggal, Sarayut Janmahasatian, Chiea-Chuen Khor, Qiao Fan, Xin Zhou, Terri L. Young, E-Shyong Tai, Liang-Kee Goh, Yi-Ju Li, Tin Aung, Eranga Vithana, Yik-Ying Teo, Wanting Tay, Xueling Sim, Igor Rudan, Caroline Hayward, Alan F. Wright, Ozren Polasek, Harry Campbell, James F. Wilson, Brian W. Fleck, Isao Nakata, Nagahisa Yoshimura, Ryo Yamada, Fumihiko Matsuda, Kyoko Ohno-Matsui, Abhishek Nag, George McMahon, Beate St. Pourcain, Yi Lu, Jugnoo S. Rahi, Phillippa M. Cumberland, Shomi Bhattacharya, Claire L. Simpson, Larry D. Atwood, Xiaohui Li, Leslie J. Raffel, Federico Murgia, Laura Portas, Dominiek D. G. Despriet, Leonieke M. E. van Koolwijk, Christian Wolfram, Karl J. Lackner, Anke Tönjes, Reedik Mägi, Terho Lehtimäki, Mika Kähönen, Tõnu Esko, Andres Metspalu, Taina Rantanen, Olavi Pärssinen, Barbara E. Klein, Thomas Meitinger, Timothy D. Spector, Ben A. Oostra, Albert V. Smith, Paulus T. V. M. de Jong, Albert Hofman, Najaf Amin, Lennart C. Karssen, Fernando Rivadeneira, Johannes R. Vingerling, Guðný Eiríksdóttir, Vilmundur Gudnason, Angela Döring, Thomas Bettecken, André G. Uitterlinden, Cathy Williams, Tanja Zeller, Raphaële Castagné, Konrad Oexle, Cornelia M. van Duijn, Sudha K. Iyengar, Paul Mitchell, Jie Jin Wang, René Höhn, Norbert Pfeiffer, Joan E. Bailey-Wilson, Dwight Stambolian, Tien-Yin Wong, Christopher J. Hammond, Caroline C. W. Klaver

**Affiliations:** 1 Department of Ophthalmology, Erasmus Medical Center, PO Box 2040, 3000 CA Rotterdam, The Netherlands; 2 Department of Epidemiology, Erasmus Medical Center, PO Box 2040, 3000 CA Rotterdam, The Netherlands; 3 Department of Twin Research and Genetic Epidemiology, King’s College London, St. Thomas’ Hospital, London, UK; 4 Saw Swee Hock School of Public Health, National University of Singapore, Singapore, Singapore; 5 Singapore National Eye Centre, Singapore Eye Research Institute, Singapore, Singapore; 6 Medical Research Council Human Genetics Unit, Institute of Genetics and Molecular Medicine, University of Edinburgh, Edinburgh, UK; 7 Department of Ophthalmology, J. Gutenberg University Medical Center, Mainz, Germany; 8 School of Optometry and Vision Sciences, Cardiff University, Cardiff, UK; 9 Division of Epidemiology and Clinical Applications, National Eye Institute, Intramural Research Program, National Institutes of Health, Bethesda, USA; 10 Department of Ophthalmology, Kyoto University Graduate School of Medicine, Kyoto, Japan; 11 Centre for Eye Research Australia, Royal Victorian Eye and Ear Hospital, University of Melbourne, Melbourne, Australia; 12 Centre for Ophthalmology and Visual Science, Lions Eye Institute, University of Western Australia, Perth, Australia; 13 Department of Epidemiology, Johns Hopkins Bloomberg School of Public Health, Baltimore, USA; 14 Inherited Disease Research Branch, National Human Genome Research Institute, National Institutes of Health, Baltimore, USA; 15 Department of Ophthalmology, National University Health System, National University of Singapore, Singapore, Singapore; 16 Department of Epidemiology and Biostatistics, Case Western Reserve University, Cleveland, USA; 17 Genome Institute of Singapore, Agency for Science, Technology and Research, Singapore, Singapore; 18 Center for Human Genetics, Duke University Medical Center, Durham, USA; 19 Department of Medicine, National University of Singapore, Singapore, Singapore; 20 Duke-National University of Singapore Graduate Medical School, Singapore, Singapore; 21 Department of Statistics and Applied Probability, National University of Singapore, Singapore, Singapore; 22 Centre for Molecular Epidemiology, National University of Singapore, Singapore, Singapore; 23 Centre for Population Health Sciences, University of Edinburgh, Edinburgh, UK; 24 Faculty of Medicine, University of Split, Split, Croatia; 25 Princess Alexandra Eye Pavilion, Edinburgh, UK; 26 Center for Genomic Medicine, Kyoto University Graduate School of Medicine, Kyoto, Japan; 27 Department of Ophthalmology and Visual Science, Tokyo Medical and Dental University, Tokyo, Japan; 28 School of Social and Community Medicine, University of Bristol, Bristol, UK; 29 Department of Genetics and Population Health, Queensland Institute of Medical Research, Brisbane, Australia; 30 Medical Research Council Centre of Epidemiology for Child Health, Institute of Child Health, University College London, London, UK; 31 Institute of Ophthalmology, University College London, London, UK; 32 Ulverscroft Vision Research Group, University College London, London, UK; 33 Department of Neurology, Boston University School of Medicine, Boston, USA; 34 Medical Genetics Institute, Cedars-Sinai Medical Center, Los Angeles, USA; 35 Institute of Population Genetics, National Research Council, Sassari, Italy; 36 Glaucoma Service, The Rotterdam Eye Hospital, Rotterdam, The Netherlands; 37 Institute of Clinical Chemistry and Laboratory Medicine, J. Gutenberg University Medical Center, Mainz, Germany; 38 Department of Medicine, University of Leipzig, Leipzig, Germany; 39 Integrated Research and Treatment Center (IFB) AdiposityDiseases, University of Leipzig, Leipzig, Germany; 40 Estonian Genome Center, University of Tartu, Tartu, Estonia; 41 The Wellcome Trust Centre for Human Genetics, University of Oxford, Oxford, UK; 42 Department of Clinical Chemistry, Fimlab Laboratories, Tampere University Hospital, Tampere, Finland; 43 University of Tampere School of Medicine, Tampere, Finland; 44 Department of Clinical Physiology, Tampere University Hospital, Tampere, Finland; 45 Department of Clinical Physiology, University of Tampere School of Medicine, Tampere, Finland; 46 Department of Health Sciences, Gerontology Research Center, University of Jyväskylä, Jyväskylä, Finland; 47 Department of Ophthalmology, Central Hospital of Central Finland, Jyväskylä, Finland; 48 Department of Ophthalmology and Visual Sciences, University of Wisconsin School of Medicine and Public Health, Madison, USA; 49 Helmholtz Zentrum München, German Research Center for Environmental Health, Institute of Epidemiology I, Neuherberg, Germany; 50 Institute of Human Genetics, Technical University Munich, Munich, Germany; 51 Department of Clinical Genetics, Erasmus Medical Center, Rotterdam, The Netherlands; 52 Department of Medicine, University of Iceland, Reykjavik, Iceland; 53 Icelandic Heart Association, Kopavogur, Iceland; 54 Department of Clinical and Molecular Ophthalmogenetics, Netherlands Institute of Neurosciences (NIN), An Institute of the Royal Netherlands Academy of Arts and Sciences (KNAW), Amsterdam, The Netherlands; 55 Department of Internal Medicine, Erasmus Medical Center, Rotterdam, The Netherlands; 56 Center for Applied Genotyping, Max Planck Institute of Psychiatry, German Research Institute of Psychiatry, Munich, Germany; 57 Centre for Child and Adolescent Health, University of Bristol, Bristol, UK; 58 Clinic for General and Interventional Cardiology, University Heart Center Hamburg, Hamburg, Germany; 59 INSERM UMRS 937, Pierre and Marie Curie University (UPMC, Paris 6) and Medical School, Paris, France; 60 Department of Ophthalmology, Centre for Vision Research, Westmead Millennium Institute, University of Sydney, Sydney, Australia; 61 Department of Ophthalmology, University of Pennsylvania, Philadelphia, USA; 63 Department of Ophthalmology, Academic Medical Center, Amsterdam, The Netherlands; 64 Helmholtz Zentrum München, German Research Center for Environmental Health, Institute of Epidemiology II, Neuherberg, Germany

## Abstract

Myopia is a complex genetic disorder and a common cause of visual impairment among working age adults. Genome-wide association studies have identified susceptibility loci on chromosomes 15q14 and 15q25 in Caucasian populations of European ancestry. Here, we present a confirmation and meta-analysis study in which we assessed whether these two loci are also associated with myopia in other populations. The study population comprised 31 cohorts from the Consortium of Refractive Error and Myopia (CREAM) representing 4 different continents with 55,177 individuals; 42,845 Caucasians and 12,332 Asians. We performed a meta-analysis of 14 single nucleotide polymorphisms (SNPs) on 15q14 and 5 SNPs on 15q25 using linear regression analysis with spherical equivalent as a quantitative outcome, adjusted for age and sex. We calculated the odds ratio (OR) of myopia versus hyperopia for carriers of the top-SNP alleles using a fixed effects meta-analysis. At locus 15q14, all SNPs were significantly replicated, with the lowest *P* value 3.87 × 10^−12^ for SNP rs634990 in Caucasians, and 9.65 × 10^−4^ for rs8032019 in Asians. The overall meta-analysis provided *P* value 9.20 × 10^−23^ for the top SNP rs634990. The risk of myopia versus hyperopia was OR 1.88 (95 % CI 1.64, 2.16, *P* < 0.001) for homozygous carriers of the risk allele at the top SNP rs634990, and OR 1.33 (95 % CI 1.19, 1.49, *P* < 0.001) for heterozygous carriers. SNPs at locus 15q25 did not replicate significantly (*P* value 5.81 × 10^−2^ for top SNP rs939661). We conclude that common variants at chromosome 15q14 influence susceptibility for myopia in Caucasian and Asian populations world-wide.

**Electronic supplementary material:**

The online version of this article (doi:10.1007/s00439-012-1176-0) contains supplementary material, which is available to authorized users.

## Introduction

Refractive errors are common optical defects of the visual system. An important refractive error is myopia (nearsightedness), which occurs when the eye elongates beyond the focal plane. The prevalence of myopia is high, affecting about one-third of the world’s population, and reaching over 70 % in certain Asian ethnic groups (He et al. 2004; Kempen et al. 2004; Lin et al. 2004; Vitale et al. 2008; Wu et al. 2001). High degrees of myopia are associated with pathologic ocular changes, such as myopic macular degeneration, retinal detachment, and glaucoma (Curtin and Karlin 1971; McBrien and Gentle 2003; Saw 2006; Saw et al. 2005; Tano 2002). Due to the limited treatment options, myopia is a common cause of visual impairment (Tano 2002; Young 2009).

Refractive errors, and myopia in particular, are complex genetic traits with a largely unknown etiology. Established environmental factors are education, early reading, and reduced outdoor exposure (Dirani et al. 2009; Ip et al. 2008; McBrien et al. 2008; Morgan and Rose 2005; Rose et al. 2008; Saw et al. 2001; Young 2009). Although heritability estimates are high [50–90 % (Young et al. 2007)], the search for myopia genes is still ongoing. Previous linkage and association studies have led to the identification of at least 18 myopia (MYP) loci, 10 additional chromosomal regions, and several candidate genes (Baird et al. 2010; Young 2009). Replication of these associations has been inconsistent, and their application to the general population is limited (Baird et al. 2010).

Recent genome-wide association studies (GWAS) reported several susceptibility loci for refractive error and myopia (Hysi et al. 2010; Li et al. 2011a, b; Nakanishi et al. 2009; Shi et al. 2011; Solouki et al. 2010). Solouki et al. (2010) and Hysi et al. (2010) were the first to perform a GWAS in a general Caucasian population, and identified susceptibility loci on chromosomes 15q14 and 15q25, respectively. In both studies, carriers of single nucleotide polymorphism (SNP) rs634990 at 15q14 (OR 1.83, 95 % CI 1.42–2.36) and of SNP rs8027411 at 15q25 (OR 1.16, 95 % CI 1.02–1.28) had a higher risk of myopia. Confirmation of these findings was obtained in various replication studies (Hayashi et al. 2011; Hysi et al. 2010; Solouki et al. 2010). However, these replication cohorts were relatively limited in size, increasing the chance of a type 1 error.

To address potential inaccuracies and to investigate generalizability, we investigated the associations between refractive error, and the 15q14 and 15q25 susceptibility loci in a large international replication and meta-analysis study (Consortium of Refractive Error and Myopia, CREAM) including 31 cohorts with various ethnicities from 4 different continents.

## Results

### Meta-analysis of allelic effects on spherical equivalent (SE)

**Fig. 1:**
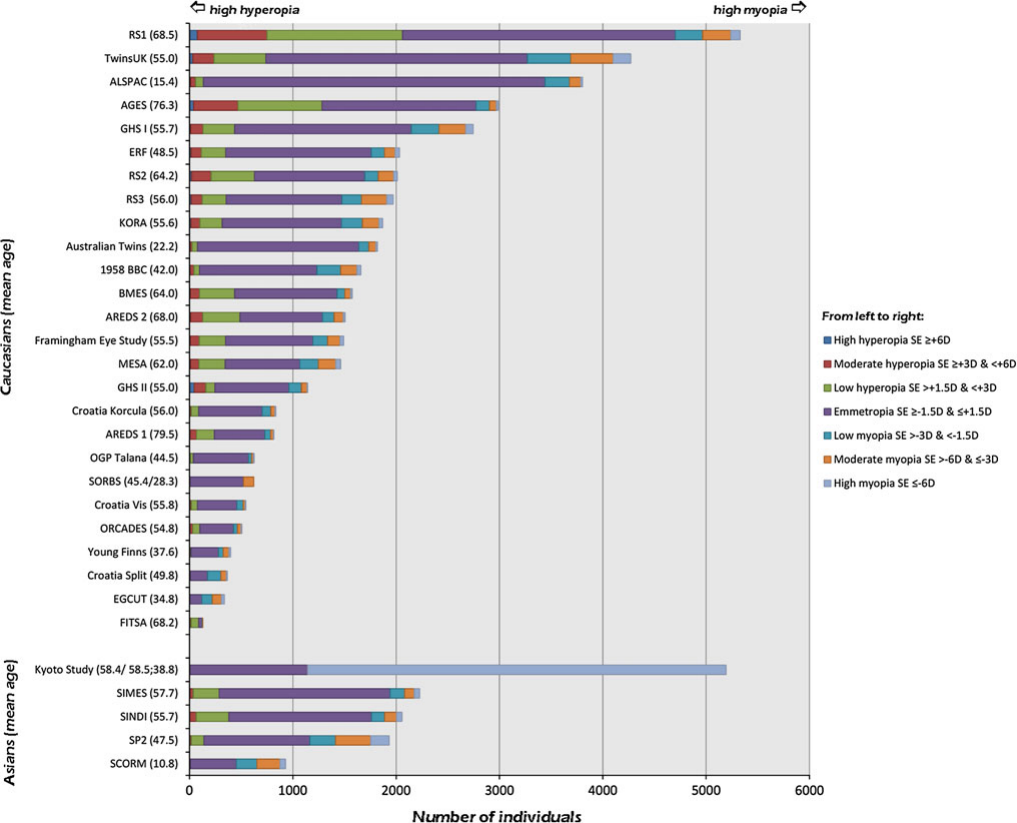
Mean age and distribution of spherical equivalent in all study cohorts

Complete data on refractive error and genome-wide SNPs were available in all 29 population-based studies comprising 49,364 subjects: 42,224 Caucasians and 7,140 Asians (Table 1; Fig. 1, Supplementary Table 1). This includes the previously reported discovery set consisting of 15,608 (Solouki et al. 2010) and 17,608 subjects (Hysi et al. 2010), respectively.

**Table 1:** Descriptives of all study cohorts

Study	*n*	Mean age (SD)	Age range	Men (%)	Mean SE (SD)
1958 British Birth Cohort	1,658	42 (0.0)	40–50	54.2	−0.96 (2.00)
AGES Reykjavik	2,986	76.3 (5.4)	60–80+	35.3	1.22 (2.05)
ALSPAC	3,804	15.4 (0.3)	14.25–17.08	47.2	−0.38 (1.28)
AREDS 1	816	79.5 (5.1)	60–80+	43.5	0.68 (1.94)
AREDS 2	1,506	68.0 (4.7)	55–81	41.1	0.54 (2.25)
Australian Twins	1,819	22.2 (12.7)	5–90	44.0	−0.22 (1.28)
Blue Mountains Eye Study	1,574	64 (7.9)	50–80+	43.4	0.59 (1.96)
Croatia Split	366	49.8 (14.4)	18–85	46.0	−1.83 (1.83)
Croatia Vis Island	544	55.8 (14.0)	18–83	40.0	−0.16 (1.93)
Croatia Korcula Island	836	56.0 (13.8)	18–98	35.0	−0.25 (1.92)
ERF	2,032	48.5 (14.3)	18+	43.1	0.07 (2.13)
EGCUT	338	34.8 (15.2)	18–85	36.9	−2.60 (2.00)
Finnish Twin Study on Aging	127	68.2 (3.8)	63–76	0.0	1.68 (1.54)
Framingham Eye Study	1,500	55.5 (9.0)	20–80	42.5	−0.17 (2.40)
Gutenberg Health Study I	2,745	55.7 (11)	35–74	51.5	−0.38 (2.44)
Gutenberg Health Study II	1,142	55.0 (10.9)	35–74	49.8	−0.41 (2.58)
KORA	1,867	55.6 (11.7)	35–84	49.6	−0.29 (2.27)
MESA	1,462	62 (9.4)	46–86	49.5	−0.28 (2.62)
ORCADES	505	54.8 (13.7)	22–88.5	43.0	0.01 (2.14)
Rotterdam Study 1	5,328	68.5 (8.6)	55+	41.3	0.86 (2.45)
Rotterdam Study 2	2,009	64.2 (7.4)	55+	45.9	0.48 (2.51)
Rotterdam Study 3	1,970	56.0 (5.5)	45+	43.9	−0.35 (2.62)
OGP Talana	623	44.5 (21.1)	5–89	51.8	−0.15 (1.78)
SCORM	929	10.8 (0.8)	10–15	48.0	−2.02 (2.26)
SiMES	2,226	57.7 (10.8)	40–80	49.3	−0.08 (1.98)
SINDI	2,055	55.7 (8.7)	40–80+	51.2	0.01 (2.13)
SP2	1,930	47.5 (10.9)	20–80	45.4	−1.67 (2.89)
TwinsUK	4,270	55.0 (12.0)	20–82	7.4	−0.39 (2.73)
Young Finns	397	37.6 (5.2)	25–50	45.0	−1.20 (2.29)
Kyoto Study	5,192	na	na	na	na
Cases	1,143	58.4 (14.3)	20–91	33.3	−10.50 (6.44)
Controls 1	3,120	58.5 (13.6)	20–90	61.7	na
Controls 2	929	38.8 (11.8)	0–74	41.3	na
SORBS	621	na	na	na	na
Cases	100	45.4 (6.6)	18–40	36.4	na
Controls	521	28.3 (15.16)	18–80	45.0	na

Table 2 shows the results of the meta-analysis of the 14 SNPs (Hysi et al. 2010; Solouki et al. 2010) at locus 15q14 and 5 SNPs at locus 15q25. The frequency of the effect allele C for top SNP rs634990 at locus 15q14 ranged from 0.38 to 0.64, while frequency of the effect allele A for top SNP rs939661 at 15q25 showed a larger variation, ranging from 0.28 to 0.63 (Supplementary Figure 1). The sample size of each SNP per study is provided in Supplementary Table 1. For locus 15q14, the magnitude and direction of the effects were consistent in all cohorts except Croatia Vis and SIMES. For locus 15q25, there was less consistency; for top SNP rs939661 8 cohorts—both Caucasian and Asian (Australian Twins, Croatia Split, Croatia Vis, EGCUT, FITSA, GHS II, ORCADES, and SIMES)—had a regression beta coefficient in the opposite direction to that of the other studies.

**Table 2:** Meta-analysis of allelic effects on spherical equivalent at locus 15q14 and 15q25

SNP	Position	Effect allele	Non effect allele	Freq.	Discovery (*n* = 15,608)^a^			Replication (*n* = 33,755)^b^			Caucasian (*n* = 26,615)^c^		
					beta	se	*P*	beta	se	*P*	beta	se	*P*
*Locus 15q14*													
rs634990	32793365	C	T	0.49	−0.23	0.03	1.35 x 10^−14^	−0.09	0.01	4.53 x 10^−14^	−0.08	0.01	3.87 x 10^−12^
rs560766	32788234	A	G	0.48	−0.20	0.03	4.82 x 10^−12^	−0.09	0.01	3.53 x 10^−14^	−0.08	0.01	3.91 x 10^−12^
rs524952	32793178	A	T	0.48	−0.23	0.03	1.19 x 10^−14^	−0.08	0.01	9.05 x 10^−13^	−0.08	0.01	1.07 x 10^−11^
rs688220	32786167	A	G	0.48	−0.20	0.03	4.43 x 10^−12^	−0.08	0.01	1.01 x 10^−13^	−0.08	0.01	1.38 x 10^−11^
rs580839	32786121	A	G	0.48	−0.20	0.03	4.39 x 10^−12^	−0.08	0.01	1.05 x 10^−13^	−0.08	0.01	1.34 x 10^−11^
rs11073060	32777143	A	C	0.48	−0.21	0.03	1.12 x 10^−12^	−0.08	0.01	2.46 x 10^−13^	−0.08	0.01	2.47 x 10^−11^
rs4924134	32781857	G	A	0.45	−0.21	0.03	1.20 x 10^−12^	−0.08	0.01	3.01 x 10^−13^	−0.08	0.01	2.96 x 10^−11^
rs7176510	32786771	T	C	0.45	−0.20	0.03	1.70 x 10^−11^	−0.09	0.01	8.31 x 10^−14^	−0.08	0.01	7.81 x 10^−12^
rs619788	32782398	A	C	0.44	−0.20	0.03	3.94 x 10^−12^	−0.08	0.01	2.21 x 10^−13^	−0.08	0.01	2.29 x 10^−11^
rs7163001	32777866	A	G	0.44	−0.21	0.03	1.26 x 10^−12^	−0.08	0.01	6.28 x 10^−13^	−0.08	0.01	4.16 x 10^−11^
rs11073059	32776966	A	T	0.44	−0.21	0.03	1.98 x 10^−12^	−0.08	0.01	8.78 x 10^−13^	−0.08	0.01	4.85 x 10^−11^
rs11073058	32776918	T	G	0.44	−0.20	0.03	2.23 x 10^−12^	−0.08	0.01	8.52 x 10^−13^	−0.08	0.01	4.84 x 10^−11^
rs685352	32795627	G	A	0.46	−0.21	0.03	4.55 x 10^−13^	−0.08	0.01	4.32 x 10^−12^	−0.08	0.01	2.09 x 10^−10^
rs8032019	32778782	G	A	0.40	−0.19	0.03	1.00 x 10^−10^	−0.08	0.01	5.81 x 10^−12^	−0.08	0.01	7.00 x 10^−10^

SNP	Position	Effect allele	Non effect allele	Freq.	Discovery (*n* = 17,806)^a^			Replication (*n* = 31,557)^b^			Caucasian (*n* = 24,417)^c^		
					beta	se	*P*	beta	se	*P*	beta	se	*P*
*Locus 15q25*													
rs939661	77218118	A	G	0.51	−0.15	0.03	3.85 x 10^−9^	−0.02	0.01	5.81 x 10^−2^	−0.02	0.01	7.73 x 10^−2^
rs939658	77238924	G	A	0.51	−0.15	0.03	1.85 x 10^−9^	−0.02	0.01	1.60 x 10^−1^	−0.02	0.01	2.16 x 10^−1^
rs17175798	77251015	C	T	0.51	−0.15	0.03	1.99 x 10^−9^	−0.02	0.01	1.81 x 10^−1^	−0.01	0.01	2.38 x 10^−1^
rs8033963	77242405	C	C	0.51	−0.15	0.03	1.86 x 10^−9^	−0.01	0.01	2.18 x 10^−1^	−0.02	0.01	2.20 x 10^−1^
rs8027411	77248084	T	G	0.51	−0.15	0.03	2.07 x 10^−9^	−0.01	0.01	2.49 x 10^−1^	−0.02	0.01	2.16 x 10^−1^

SNP	Position	Effect allele	Non effect allele	Freq.	Asian (*n* = 7,140)^d^			Meta-analysis (*n* = 49,363)^e^		
					beta	se	*P*	beta	se	*P*
*Locus 15q14*										
rs634990	32793365	C	T	0.49	−0.12	0.04	2.21 x 10^−3^	−0.11	0.01	9.20 x 10^−3^
rs560766	32788234	A	G	0.48	−0.12	0.04	1.47 x 10^−3^	−0.10	0.01	1.03 x 10^−21^
rs524952	32793178	A	T	0.48	−0.18	0.07	9.52 x 10^−3^	−0.10	0.01	2.00 x 10^−21^
rs688220	32786167	A	G	0.48	−0.12	0.04	9.80 x 10^−4^	−0.10	0.01	3.44 x 10^−21^
rs580839	32786121	A	G	0.48	−0.12	0.04	1.10 x 10^−3^	−0.10	0.01	3.51 x 10^−21^
rs11073060	32777143	A	C	0.48	−0.12	0.04	1.45 x 10^−3^	−0.10	0.01	5.13 x 10^−21^
rs4924134	32781857	G	A	0.45	−0.12	0.04	1.60 x 10^−3^	−0.10	0.01	5.57 x 10^−21^
rs7176510	32786771	T	C	0.45	−0.12	0.04	1.74 x 10^−3^	−0.10	0.01	6.09 x 10^−21^
rs619788	32782398	A	C	0.44	−0.12	0.04	1.54 x 10^−3^	−0.10	0.01	6.97 x 10^−21^
rs7163001	32777866	A	G	0.44	−0.11	0.04	2.81 x 10^−3^	−0.10	0.01	1.41 x 10^−20^
rs11073059	32776966	A	T	0.44	−0.11	0.04	3.64 x 10^−3^	−0.10	0.01	2.63 x 10^−20^
rs11073058	32776918	T	G	0.44	−0.11	0.04	3.50 x 10^−3^	−0.10	0.01	2.68 x 10^−20^
rs685352	32795627	G	A	0.46	−0.11	0.04	4.14 x 10^−3^	−0.10	0.01	8.10 x 10^−20^
rs8032019	32778782	G	A	0.40	−0.13	0.04	9.65 x 10^−4^	−0.10	0.01	1.78 x 10^−18^
*Locus 15q25*										
rs939661	77218118	A	G	0.51	−0.03	0.04	4.86 x 10^−1^	−0.04	0.01	1.22 x 10^−4^
rs939658	77238924	G	A	0.51	−0.04	0.05	3.94 x 10^−1^	−0.04	0.01	4.32 x 10^−4^
rs17175798	77251015	C	T	0.51	−0.05	0.06	3.70 x 10^−1^	−0.04	0.01	6.12 x 10^−4^
rs8033963	77242405	C	C	0.51	−0.01	0.04	8.42 x 10^−1^	−0.04	0.01	9.37 x 10^−4^
rs8027411	77248084	T	G	0.51	0.00	0.04	9.12 x 10^−1^	−0.03	0.01	1.14 x 10^−3^

*Freq* average frequency

^a^For the 15q14 locus: RS1, RS2, RS3, ERF, TwinsUK; for the 15q25 locus: TwinsUK, RS1, RS2, RS3, ERF, 1958 British Birth Cohort, Australian Twins (adult samples only)

^b^For the 15q14 locus: 1958 British Birth Cohort, AGES, ALSPAC, AREDS 1, AREDS 2, Australian Twins, BMES, Croatia Split, Croatia Vis, Croatia Korcula, EGCUT, FITSA, Framingham, GHS I, GHS II, KORA, MESA, ORCADES, OGP Talana, SCORM, SiMES, SINDI, SP2, Young Finns; for the 15q25 locus: AGES, ALSPAC, AREDS 1, AREDS 2, BMES, Croatia Split, Croatia Vis, Croatia Korcula, EGCUT, FITSA, Framingham, GHS I, GHS II, KORA, MESA, ORCADES, OGP Talana, Young Finns, SCORM, SiMES, SINDI, SP2

^c^For the 15q14 locus: 1958 British Birth Cohort, AGES, ALSPAC, AREDS 1, AREDS 2, Australian Twins, BMES, Croatia Split, Croatia Vis, Croatia Korcula, EGCUT, FITSA, Framingham, GHS I, GHS II, KORA, MESA, ORCADES, OGP Talana, Young Finns; for 15q25 locus: AGES, ALSPAC, AREDS 1, AREDS 2, BMES, Croatia Split, Croatia Vis, Croatia Korcula, EGCUT, FITSA, Framingham, GHS I, GHS II, KORA, MESA, ORCADES, OGP Talana, Young Finns

^d^Asian replication: SP2, SIMES, SINDI, SCORM

^e^All studies

For locus 15q14, the replication set, consisting of all studies except the ones previously used in the discovery analysis, showed a statistically significant association between SE and all SNPs with a best *P* value 4.53 × 10^−14^ for top SNP rs634990. Confirmation was achieved in 23 out of 25 Caucasian studies (overall *P* 3.87 × 10^−12^ for SNP rs634990), and in 3 out of 4 Asian studies (overall *P* 2.21 × 10^−3^ for SNP rs634990). Meta-analysis of the discovery and replication cohorts together provided *P* value 9.20 × 10^−23^ for SNP rs634990.

For locus 15q25, neither Caucasian nor Asian validation studies replicated the original association. Meta-analysis of the combined set of the 5 SNPs yielded a lowest *P* 1.22 × 10^−4^ for SNP rs939661. As a subsequent analysis, we investigated locus 15q25 in more detail, and tested another 26 SNPs in 26 out of 29 cohorts (no data available in ALSPAC, AREDS 1, and EGCUT). This set of SNPs was not replicated either, however, meta-analysis including the discovery cohort was still significant (best *P* 2.07 × 10^−4^ for SNP rs1915726; Supplementary Table 3).

### Meta-analysis of risk of myopia for top SNP

**Fig. 2:**
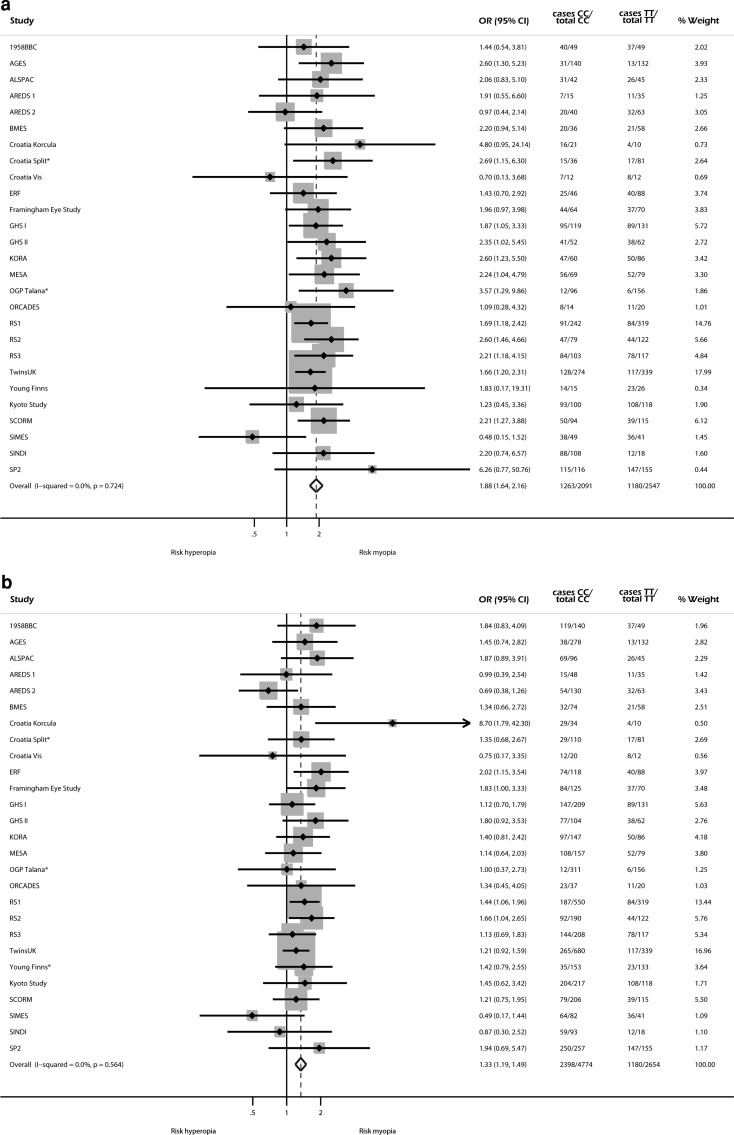
Forest plots of odds ratios of myopia (spherical equivalent ≤−3 diopters) versus hyperopia (spherical equivalent ≥+3 diopters) for top SNP rs634990. *For studies without subjects with high or moderate hyperopia, emmetropia was used as a reference group. **a** Homozygotes carriers of alleles TT versus CC for SNP rs634990. **b** Heterozygotes carriers of alleles TT versus TC for SNP rs634990

Genotype distributions for rs634990 at locus 15q14 were available for 28 out of 31 studies (all but FITSA, Australian Twins, and SORBS). There was no evidence of heterogeneity in the analyses of homozygote carriers [χ^2^ 21.35 (d.f. 26), *P* 0.724, *I* ^2^ 0.0 %] or heterozygote carriers [χ^2^ 24.22 (d.f. 26), *P* 0.564, *I* ^2^ 0.0 %]. Therefore, only results from fixed effects meta-analysis were used. Figure 2 shows the forest plots for the risk of myopia for homozygous and heterozygous carriers of the top SNP rs634990. The OR of moderate to high myopia (SE ≤−3 D) versus moderate to high hyperopia (SE ≥+3 D) was 1.88 (95 % CI 1.64, 2.16, *P* < 0.001) for homozygous carriers of the risk allele at the top SNP rs634990, and 1.33 (95 % CI 1.19, 1.49, *P* < 0.001) for heterozygous carriers.

## Discussion

Chromosome 15q was first implicated in refractive error and myopia by genome-wide analysis of two large studies located in Northern Europe (Hysi et al. 2010; Solouki et al. 2010). Here, in an international meta-analysis consisting of 31 independent studies from the CREAM consortium, we provide further support that the association with locus 15q14 is robust and present in both Caucasians and Asians. We combined the results with those of the initial study into a powerful meta-analysis of highly associated SNPs with a total study population of 55,177 participants. The combined results showed that all tested SNPs for locus 15q14 were associated with refractive errors, and that homozygous carriers of the top SNP rs634990 had approximately twice the risk of myopia. SNPs at the other locus, 15q25, could not be convincingly replicated.

This study has strengths and limitations. Major strengths of the study include the sample size and the inclusion of different ethnicities. The CREAM consortium represents the largest study on refractive error known to date. Previous replication studies have not been large scaled and focused on populations of the same ancestry (Gao et al. 2012; Lu et al. 2011; Wang et al. 2011). Another advantage of our study is the incorporation of clinical relevant endpoints such as high myopia and high hyperopia. Among the limitations are differences in designs and methods of the studies. (1) Population-based as well as case control studies were incorporated. However, the latter were only two (Kyoto Study and SORBS) and both had results within the same range as the population-based studies. (2) Different types of equipment and measurement methods were used to detect refractive error. These differences are generally subtle, and are not likely to cause false findings. (3) Various methods of genotyping and imputation were used, and genotyping was not complete in all studies. All SNPs at 15q14 had similar effect; thus, we do not think this has influenced these associations. SNPs at 15q25 showed larger variation, and the incomplete genotyping may have underpowered this analysis.

Earlier replication of the 15q14 locus was reported by Hayashi et al. (2011) in a Japanese sample of high myopic probands and controls. In a comparison of 1,125 high myopes (axial length >26.1 mm) versus 1,295 controls, the risk of high myopia was increased for the carriers of the initial top SNP rs634990 [OR 1.84 in homozygotes (95 % CI 1.44–2.36)]. Taken together with the current findings, this suggests that 15q14 plays a role in both common and high myopia.

The 15q14 associated region contains two interesting genes that are both well expressed in the retina, *GJD2* and *ACTC1. GJD2* encodes the Connexin36 protein, which plays a crucial role in the transmission and processing of visual signals in the retina by enabling intercellular transport of small molecules and ions in photoreceptors, amacrine and bipolar cells (Deans et al. 2002; Guldenagel et al. 2001; Kihara et al. 2009; Striedinger et al. 2005). We speculated that the protein encoded by the other candidate gene, *ACTC1*, could play a role in scleral remodeling, given the fact that similar actin proteins have been shown to be increased in developing myopic tree shrew eyes (Jobling et al. 2009). Previous *GJD2* (Solouki et al. 2010) and *ACTC1* (unpublished data) direct sequencing experiments did not reveal a functional variant, but the 15q14 locus appeared to harbor regulatory elements which may influence transcription of these genes (Solouki et al. 2010).

The 15q25 region contains the interesting candidate gene *RASGRF1*, which is highly expressed in the retina and has previously been implicated in photoreception and visual sensory processes (Fernandez-Medarde et al. 2009; Jones and Moses 2004). The association with this locus and gene is not robust, since none of the initial SNPs replicated significantly, and determination of more SNPs did not increase significance. A type 1 error may explain the initial finding. Another potential cause for the non-replication is a large variation in allele frequencies. The range of allele frequencies at 15q25 (0.28–0.63) was only slightly larger than at 15q14 (0.38–0.64) in our consortium, making this an unlikely explanation (Supplementary Figure 1). Finally, population stratification within cohorts did not appear to play a major role, since only two cohorts had significant principal components, which were addressed in the analyses.

Other GWAS loci were only found for high myopia in Asian case control studies, and they were located on chromosomes 11q24.1 (Nakanishi et al. 2009), 5p15 (Li et al. 2011a), 4q25 (Li et al. 2011b), and 13q12.12 (Shi et al. 2011). The locus on chromosome 5p15 harbors the excellent candidate gene *CTNND2* which is involved in retinal morphogenesis, adhesion, retinal cell architecture integrity (Duparc et al. 2006; Paffenholz et al. 1999), and was replicated in subjects of the same ethnicity (Lu et al. 2011). Replication studies for the 4q25 (Gao et al. 2012) and 11q24.1 (Wang et al. 2011) loci were only successful in case of the 4q25 locus; these loci did not have prominent candidate genes.

What should be the next steps? For 15q14, comprehensive resequencing of the entire associated region and the flanking genes can reveal the responsible gene defects which determine the association. Novel techniques such as next-generation sequencing are promising in this regard. Functional studies in knockout animals will shed light on potential protein effects. Finally, evaluation of gene-environment interactions may explain phenotypic variation and help identify high risk groups. For myopia genetics in general, performance of a genome-wide meta-analysis is a logical next step. The current CREAM collaboration is an excellent platform for this project.

In summary, we have convincingly demonstrated that common variants at chromosome 15q14 influence susceptibility for myopia in both Caucasian and Asian populations around the world. Identification of functional variants and responsible genes that explain this association will provide more insight in the complex etiology of myopia.

## Materials and methods

### Subjects and phenotyping

A total of 31 study cohorts from the Consortium of Refractive Error and Myopia (CREAM) participated in this meta-analysis. 29 population-based as well as 2 case–control studies were included. General methods, descriptives and phenotyping and genotyping methods of the study cohorts can be found in Table 1, the Supplementary Material and Supplementary Table 1, respectively. In short, 22 cohorts consisted of Caucasian, and 5 of Asian study subjects. All studies were performed with the approval of their local Medical Ethics Committee, and written informed consent was obtained from all participants in accordance with the Declaration of Helsinki.

All studies used a similar protocol for phenotyping. Exclusion criteria were age ≤10 years, and bilateral cataract surgery, laser refractive procedures or other intra-ocular procedures which might alter refraction. Eligible participants underwent a complete ophthalmologic examination including a non-dilated measurement of refractive error (Table 1) of both eyes. Spherical equivalent was calculated according to the standard formula (SE = sphere + ½ cylinder), and the mean of two eyes was used for analysis. When data from only one eye were available, the SE of this eye was used. SE was categorized into low (SE from −1.5 to −3 D), moderate (SE from −3 to −6 D) and high (SE of −6 D or lower) myopia; and also into low (SE from +1.5 to +3 D), moderate (SE from +3 to +6 D) and high (SE of +6 D or higher) hyperopia. Emmetropia was defined as SE equal to or between −1.5 and +1.5 D.

### Genotyping and imputation

DNA was extracted according to standard procedures, and genotyping and imputation of SNPs across the entire genome was performed using various methods (Table 1). Samples with a low call rate, with excess autosomal heterozygosity, with sex-mismatch, or outliers identified by the identity-by-state clustering analysis were excluded.

### Statistical analysis

#### Meta-analysis of allelic effects on spherical equivalent

We selected 19 SNPs within loci 15q14 (14 SNPs) and 15q25 (5 SNPs) with a *P* value of <10^−6^ from two previous GWAS (Hysi et al. 2010; Solouki et al. 2010). Linear regression models with a 1 degree of freedom trend test were used to examine associations with SE as a quantitative trait outcome, adjusting for age and gender and significant principal components if applicable. From all population-based cohorts, we obtained effect allele, non effect allele, regression coefficient beta, standard error, *P* value, minor allele and minor allele frequency for each of these SNPs. METAL for Linux was used to perform a meta-analysis on betas and standard errors for all SNPs. First, discovery cohorts (Hysi et al. 2010; Solouki et al. 2010) and replication studies were analyzed separately, followed by a combined meta-analysis. As a second analysis, 26 additional SNPs within the same linkage disequilibrium (LD) block were selected and tested for association using the procedures mentioned above. For these analyses, Bonferroni corrected *P* values (0.05/number of tested SNPs) of 3.57 × 10^−3^ for 15q14, and 1.0 × 10^−2^ (5 SNPs, Table 2) or 1.92 × 10^−3^ (26 SNPs, Table 3 Supplementary Material) for 15q25 were considered statistically significant.

#### Meta-analysis of risk of myopia for top SNP

From all population-based and case control studies, we obtained genotype distributions of the replicated top SNPs. We calculated heterogeneity (χ^2^, *I* ^2^ calculated and corresponding *P* values) between studies, crude OR with corresponding 95 % CI and *P* value of moderate and high myopia versus moderate and high hyperopia with a random as well as fixed effects meta-analysis using Stata 11. When these analyses provided similar outcomes, data from fixed effect analysis were used. For studies without subjects with high or moderate hyperopia, emmetropia was used as a reference group. A standard *P* value of <0.05 was considered statistically significant.

## Electronic supplementary material

Below is the link to the electronic supplementary material.

Supplementary material 1 (DOCX 155 kb)
