# Supplementary material for: Large scale international replication and meta-analysis study confirms association of the 15q14 locus with myopia. The CREAM consortium
Source: Hum Genet. 2012 Jun 5;131(9):1467–80. doi: 10.1007/s00439-012-1176-0 (PMC3418496; doi:10.1007/s00439-012-1176-0)
Supplement: Supplementary file 1 — Supplementary material 1 (DOCX 155 kb) [file 439_2012_1176_MOESM1_ESM.docx]

**Supplementary material**

**Subjects and genotyping**

***1958 British Birth Cohort***

The 1958 British Birth Cohort (Rahi et al. 2011) is a prospective population-based cohort study that initially included 17,000 newborn children whose birth was within the first week of March 1958. All participants gave informed written consent to participate in genetic association studies, and the study was approved by the South East MultiCentre Research Ethics Committee (MREC) and the Oversight Committee for the biomedical examination of the British 1958 British birth cohort. Biomedical examination protocols were approved by the South East MREC. Assessment of refraction was undertaken in a random subsample of cohort members through non-cycloplegic autorefraction (Nikon Retinomax 2) of both eyes of each subject.
Illumina’s Human1M-Duo chip was used for genotyping. Imputation was calculated with reference to HapMap release 22 CEU population data using IMPUTE version 2. Individuals were checked for genotyping success rate (all exceeded 99%), excess or low heterozygosity (all participating subjects were checked and found within the pre-defined interval of 0.2-04). SNPs were included in the analysis if they had a genotype success rate of at least 0.95, were within Hardy-Weinberg equilibrium (p>10^-04^) and had a minor allele frequency of 0.04 or above.

***Age Gene/Environment Susceptibility – Reykjavik Study (AGES)***

The Age Gene/Environment Susceptibility – Reykjavik Study is a prospective study which examined, between 2002 and 2006, 5,764 survivors from the Reykjavik cohort (19,381 persons enrolled from a random sample of 30,795 men and women born in 1907-1935 and living in Reykjavik in 1967). Details of the study are described elsewhere (Harris et al. 2007). Participants (mean age 76 years; 43% male; 100% Caucasian) underwent a comprehensive battery of tests to provide detailed phenotypes of the cardiovascular, musculoskeletal, and neurocognitive systems as well as body composition, metabolic regulation, and sensory function. Refractive error was assessed in both eyes separately using a NIDEK ARK760A autorefractor without pharmacologic cycloplegia. The study has the approval of the Icelandic National Bioethics Committee, VSN: 00-063.
DNA, extracted from blood leukocytes using standard procedures, was genotyped in 3,660 participants using the Illumina 370CNV BeadChip array. Samples were excluded from the dataset based on sample failure, genotype mismatch with reference panel, and sex mismatch resulting in 3,219 individuals with high quality genotypes. A total of 2,986 participants had data available on genotyping and refractive error. Quality control filters at the participant level included call rate>97%, heterozygosity, and number of Mendelian errors per individual. Quality control filters for each SNP included call rate >97%, minor allele frequency >0.01, Hardy-Weinberg equilibrium p > 1x10^-6^, and differential missingness by outcome or genotype (mishap test) in PLINK <http://pngu.mgh.harvard.edu/purcell/plink> P > 1x10^-9^. We utilized the Markov Chain Haplotyping (MaCH) package (<http://www.sph.umich.edu/csg/abecasis/MACH>) 15 version 1.0.16 software; imputed to plus strand of NCBI build 36, HapMap release #22. Imputation reliability was estimated as the ratio of the empirically observed dosage variance to the expected binomial dosage variance (O/E ratio) for each SNP.

***ALSPAC* - Avon Longitudinal Study of Parents and Children**ALSPAC, also known as the ‘Children of the nineties’ study, is a prospective population-based cohort study of childhood health and well-being (Golding et al. 2001). Pregnant women with an expected date of delivery between 1st April 1991 and 31st December 1992, resident in the former Avon health authority area in Southwest England, were eligible to participate in the study. A cohort of 14,541 pregnant women was established resulting in 13,988 children who were alive at 12 months of age. Data collection has been via various methods including self-completion questionnaires sent to the mother, to her partner and after age 5 to the child; direct assessments and interviews in a research clinic. Biological samples including DNA have been collected for 10,121 of the children. For the current analysis, 4988 subjects with phenotype information at age 15 years were available. Ethical approval for the study was obtained from the ALSPAC Law and Ethics committee and the three local research-ethics committees. This research adhered to the tenets of the Declaration of Helsinki. Genotyping was performed using the Illumina HumanHap550-Quad bead array by 23andMe at either the Wellcome Trust Sanger Institute, Cambridge, UK, or the Laboratory Corporation of America, Burlington, NC, USA. Samples of known non-European ancestry, with excessive missingness (>3%), minimal or excessive autosomal heterozygosity (Sanger: <0.320 or >0.345; LabCorp: <0.310 or >0.330), cryptic relatedness (>10% IBD) or with a sex-mismatch were excluded. EIGENSTRAT analysis and multidimensional scaling analysis seeded with individuals from HapMap phase 2 revealed no additional outliers. No genomic control correction was used, since GWAS analyses for a range of traits using this dataset have shown minimal evidence of population stratification (lambda 1.01) (Medland et al. 2010). There was a total of 3800 individuals with phenotype and genotype information available, and who passed all quality control filters. SNPs with call rate <95%, minor allele frequency <1%, or Hardy-Weinberg P value ≤5 x 10^−7^ were excluded. Markov Chain Haplotyping (MACH) v 1.0.16 was used to impute unobserved marker genotypes, with HapMap CEU build 36, release 22, genotypes as the reference set. Imputed SNP were required to have an imputation reliability score of R-sqr >0.3.

***AREDS 1-2***

The Age-Related Eye Disease Study (AREDS) was initially designed as a long-term multicenter, prospective study of the clinical course of age-related macular degeneration (AMD) and age-related cataract (Age-Related Eye Disease Study Research 2001b). In addition to collecting natural history data, AREDS included a randomized clinical trial of high-dose vitamin and mineral supplements for AMD and a clinical trial of high-dose vitamin supplements for cataract (Age-Related Eye Disease Study Research 2001a, b; Clemons et al. 2003). Prior to study initiation, the protocol was approved by an independent data and safety monitoring committee and by the institutional review board for each clinical center. Written informed consent was obtained from all participants before enrollment in accordance with the Declaration of Helsinki. AREDS participants were 55 to 80 years of age at enrollment and had to be free of any illness or condition that would make long-term follow-up or compliance with study medications unlikely or difficult. On the basis of fundus photographs graded by a central reading center, best-corrected visual acuity and ophthalmologic evaluations, 4,757 participants were enrolled in one of several AMD categories, including persons with no AMD (control group). Visual acuity measurement of all participants was performed with the Electronic Visual Acuity Tester (EVA) using the Electronic ETDRS (E-ETDRS) Visual Acuity Testing Protocol. This protocol is the standard procedure developed for the Early Treatment of Diabetic Retinopathy Study (ETDRS) and adapted for AREDS. A refraction measurement was performed for participants with visual acuity of less than 74 letters in each eye at the initial visit and all participants at the randomization visit. For the current analysis, 816 participants aged 60 and older were included from the AREDS 1 population and 1506 from the AREDS 2 population. Refractive error measured by a refraction protocol at baseline enrollment into the AREDS study (Age-Related Eye Disease Study Research 1999, 2001a, b; Clemons et al. 2003) was analyzed, taking the mean measured spherical equivalent (SE) across both eyes (or SE in a single eye when both eyes were not measured) as the trait of interest. Age, gender and the first three principal components (to adjust for significant population stratification) were also included as covariates. DNA was extracted from cell lines according to standard protocols.

For AREDS 1 and 2, all participants were genotyped at the Center for Inherited Disease Research. For AREDS1, three chips were used for this genotyping: Affymetrix 100K, Illumina 100K and Illumina 300K. The requested SNPs were abstracted from each of the chips and genotypes on more than one chip were checked to ensure that the calls were the same. Principal components analysis was used to examine population substructure and individuals not of Caucasian descent were removed.
For AREDS 2, a genome-wide association study of refractive error using the Illumina 2.5M chip was performed using a subset of the control group from the original AREDS study. These control individuals are all Caucasian, do not have age-related macular degeneration (AMD) and were further screened to also exclude individuals with cataracts, retinitis pigmentosa, color blindness, other congenital eye problems, LASIK, artificial lenses, and other eye surgery. For AREDS2, genotyping of SNPs was performed using the Illumina HumanOmni2.5-4v1_B chip array.
For both studies, samples with low call rate (<98%), with low mean confidence scores over all non-missing genotypes, with chromosome anomalies, or with sex-mismatch were excluded. No samples exhibited excess heterozygosity rates (1.5 interquartile ranges above or below the upper/lower quartile ranges). Cryptic relatedness was detected by estimating IBD sharing and kinship coefficients among all possible pairs and one member of each pair exhibiting a sibling or closer relationship was dropped from the analysis. SNPs were dropped from the analysis if they exhibited more than 1 blind duplicate error, more than 1 HapMap control error or more than 1 error in HapMap control trios, a genotype call rate < 99%, minor allele frequency < 0.01, or Hardy-Weinberg P value < 10^-4^. Tests for batch effects were not significant. No sex-specific differences in allelic frequency (>0.2) or heterozygosity (>0.3) were detected. Eigenstrat was used to detect population stratification; the first 3 principal components were significant and included in all analyses. Estimates of the genomic control inflation factor were calculated after including these 3 PCs in the analyses and no additional stratification was evident (lambda=1.005). A subset of the retained SNPs was used for imputation with the Markov Chain Haplotyping (MACH) package version 1.0.17 software (imputed to plus strand of NCBI build 36, HapMap release #22; see URLs). For each imputed SNP, a reliability of imputation was estimated as the ratio of the empirically observed dosage variance to the expected binomial dosage variance (O/E ratio). PLINK was used to perform the association analyses. Genotype data from AREDS 1 and 2 are publicly available through the database of Genotype and Phenotype under the name of either the MMAP study or the AREDS study.

***Australian Twin Eye Study***

The Australian Twin Eye Study comprises participants examined as part of the Twins Eye Study in Tasmania or the Brisbane Adolescent Twins Study. Details of the study are described elsewhere (Mackey et al. 2009). Ethical approval was obtained from the Royal Victorian Eye and Ear Hospital, the University of Tasmania, the Australian Twin Registry and the Queensland Institute of Medical Research. In all subjects post-cycloplegic (following instillation of tropicamide 1%) refraction for both eyes was measured using a Humphrey-598 automatic refractor (Carl Zeiss Meditec, Inc., Miami, Florida, USA).
DNA was extracted from blood leucocytes according to standard procedures. The Australian cohorts were genotyped on the Illumina Human Hap610 Quad array. SNPs with a genotype success rate of 0.95 or above was required for inclusion of the SNP into further steps of the analysis. Only SNPs in Hardy-Weinberg equilibrium were processed: the HWE inclusion threshold was P>10x10^-6^. The minimum minor allele frequency required for inclusion of individual SNPs was 0.01. Imputation was calculated with reference to HapMap release 22 CEU using MACH (<http://www.sph.umich.edu/csg/abecasis/MACH/>).
Association analysis was performed using Merlin (<http://www.sph.umich.edu/csg/abecasis/merlin/>) in the Australian twin data. Ancestry for these individuals was determined initially through self-reporting and was verified through Principal Component decomposition of their ancestry with and without comparison with HapMap phase 2 standard populations.

***Blue Mountains Eye Study (BMES)***

The Blue Mountains Eye Study (BMES) is a population-based cohort of a predominantly white population in west of Sydney, Australia. At baseline (1992-94), 3,654 permanent residents aged 49 years or older participated (participation rate of 82.4% (Mitchell et al. 1995). During 1997-99 (BMES II A), 2,335 participants (75.1% of survivors) returned for examinations after 5 years. During 1999-2000, 1,174 (85.2%) new participants took part in an Extension Study of the BMES (BMES IIB). BMES cross-section II thus includes BMES IIA (66.5%) and BMES IIB (33.5%) participants (n=3,509) (Foran et al. 2003). From the BMES cross section II who had blood samples collected, DNA was extracted for 3,189 (90.1 %) participants. Over 98% of BMES participants were European ancestry. All BMES examinations were approved by the Human Ethics Committees of the Western Sydney Area Health Service and University of Sydney. Signed informed consent was obtained from participants at each examination.
Participants of the BMES cross section II who had DNA available in early 2009

(n=2983) were genotyped using the Illumina Human 670-Quadv1 custom genotyping array

at the Wellcome Trust Sanger Institute, Cambridge as part of WTCCC2, and 2761 had genotyping data available. Following exclusion through GWAS and DNA quality control and removal of individuals who had undergone cataract surgery, had severe visual impairment or had any known ocular pathologies such as macular degeneration and nuclear cataracts resulted in genotyping data being available for 1,574 individuals.
Imputation was performed from the 1000 Genomes using IMPUTE2.034. Imputed SNPs were excluded from the analysis when failing one or more of the following QC filters: 1) prop info ≥ 0.5 (a software-specific statistic from IMPUTE); 2) Hardy-Weinberg *P*-value < 1×10^-6^. We did not filter the SNPs with MAF < 0.01 from the imputed SNPs so that rare SNPs were included for association assessment.

***CROATIA-Split Study***

The CROATIA-Split study, Croatia, is a population-based, cross-sectional study in the Dalmatian City of Split that includes 1000 examinees aged 18-95. The study received approval from relevant ethics committees in Scotland and Croatia and followed the tenets of the Declaration of Helsinki. Non-cycloplegic autorefraction were measured on each eye using a NIDEK Ark30 hand-held autorefractometer. Measures on eyes with a history of trauma, intra-ocular surgery or LASIK operations were removed and the analysis was done on the right eye measures, unless the left eye had more complete measurements (e.g. due to trauma or cataract surgery on the right eye) (Vitart et al. 2010b). Extreme values (lying more than 3 interquartile range from the upper or lower quartile) were removed. 366 data points with good quality genotypes were used in this analysis. Inverse normal transformed spherical equivalent refraction adjusted for age and age squared was used as outcome in the genetic association analysis and obtained using the rank transformation function in GenABEL (<http://www.genabel.org/>). Genome-wide association analysis was performed using the PProbABEL package using an additive SNP allelic effect model and correcting for individual relatedness using the polygenic and mmscore functions implemented in the GenABEL package.

***CROATIA-Vis Island Study***

The CROATIA-Vis island study, Croatia, is a population-based, cross-sectional study including adult participants, aged 18–93 years (mean = 56), a subset of which (N=640) underwent a complete eye examination in summer 2007 and provided their ophthalmologic history (Vitart et al. 2010a). The study received approval from relevant ethics committees in Scotland and Croatia and followed the tenets of the Declaration of Helsinki. Non-cycloplegic autorefraction were measured on each eye using a NIDEK Ark30 hand-held autorefractometer. Measures on eyes with a history of trauma, intra-ocular surgery or LASIK operations were removed and the analysis was done on the right eye measures, unless the left eye had more complete measurements (e.g. due to trauma or cataract surgery on the right eye). Extreme values (lying more than 3 interquartile range from the upper or lower quartile) were removed. After phenotypic and genotypic quality control steps, 544 measures were available for the genetic association analysis. Inverse normal transformed spherical equivalent refraction adjusted for age and age squared was used as outcome in the genetic association analysis and obtained using the rank transformation function in the R package GenABEL . Genome-wide association analysis was performed using the PProbABEL package (<http://www.genabel.org/>) using an additive SNP allelic effect model and correcting for individual relatedness using the polygenic and mmscore functions implemented in the GenABEL package.

***CROATIA-Korcula Study***

The CROATIA-Korcula study, Croatia, is a population-based, cross-sectional study that includes a total of 969 adult examinees, aged 18-98 (mean=56.3), and most (N=930) underwent a complete eye examination (Vitart et al. 2010a). The study received approval from relevant ethics committees in Scotland and Croatia and followed the tenets of the Declaration of Helsinki. Non-cycloplegic autorefraction were measured on each eye using a NIDEK Ark30 hand-held autorefractometer. Measures on eyes with a history of trauma, intra-ocular surgery or LASIK operations were removed and the analysis was done on the right eye measures, unless the left eye had more complete measurements (e.g. due to trauma or cataract surgery on the right eye). Extreme values (lying more than 3 interquartile range from the upper or lower quartile) were removed. After phenotypic and genotypic quality control steps, 836 measures were available for the genetic association analysis. Inverse normal transformed spherical equivalent refraction adjusted for age and age squared was used as outcome in the genetic association analysis and obtained using the rank transformation function in GenABEL (<http://www.genabel.org/>).Genome-wide association analysis was performed using the PProbABEL package using an additive SNP allelic effect model and correcting for individual relatedness using the polygenic and mmscore functions implemented in the GenABEL package.

***Erasmus Rucphen Family Study (ERF)***The Erasmus Rucphen Family (ERF) Study is a family-based cohort in a genetically isolated population in the southwest of the Netherlands with over 3,000 participants aged between 18 and 86 years. Cross-sectional examination took place between 2002 and 2005. The rationale and study design of this study have been described elsewhere (Aulchenko et al. 2004; Pardo et al. 2005). Cross-sectional examination took place between 2002 and 2005, including a non-dilated automated measurement of refractive error using a Topcon RM-A2000 autorefractor. All measurements in these studies were conducted after the Medical Ethics Committee of the Erasmus University had approved the study protocols and all participants had given a written informed consent in accordance with the Declaration of Helsinki.
DNA was genotyped on one of four different platforms (Illumina 6k, Illumina 318K, Illumina 370K and Affymetrix 250K). Samples with low call rate (<97.5%), with excess autosomal heterozygosity (>0.336), or with sex-mismatch were excluded, as were outliers identified by the identity-by-state clustering analysis (outliers were defined as being >3 s.d. from population mean or having identity-by-state probabilities >97%). GWAS analyses were performed using GRIMP(Estrada et al. 2009). We used genomic control to obtain optimal and unbiased results and applied the inverse variance method of each effect size estimated for both autosomal SNPs that were genotyped and imputed in both cohorts. A set of genotyped input SNPs with call rate >98%, with minor allele frequency >0.01, and with Hardy-Weinberg P value >10^−6^ was used for imputation. We used the Markov Chain Haplotyping (MACH) package version 1.0.15 software (Rotterdam, The Netherlands; imputed to plus strand of NCBI build 36, HapMap release #22) for the analyses. For each imputed SNP, a reliability of imputation was estimated as the ratio of the empirically observed dosage variance to the expected binomial dosage variance (O/E ratio).

***Estonian Genome Center, University of Tartu (EGCUT)***

The Estonian cohort is from the population-based biobank of the Estonian Genome Project of University of Tartu (EGCUT). The whole project is conducted according to the Estonian Gene Research Act and all participants have signed the broad informed consent (<http://www.biobank.ee>, (Nelis et al. 2009)). The current cohort size is over 51,515, from 18 years of age and up, which reflects closely the age distribution in the adult Estonian population. Subjects are recruited by the general practitioners (GP) and physicians in the hospitals were randomly selected from individuals visiting GP offices or hospitals. Each participant filled out a Computer Assisted Personal interview during 1-2 hours at a doctor’s office, including personal data (place of birth, place(s) of living, nationality etc.), genealogical data (family history, three generations), educational and occupational history and lifestyle data (physical activity, dietary habits, smoking, alcohol consumption, women’s health, quality of life). Anthropometric and physiological measurements were also taken.. All diseases are defined according to the ICD10 coding. All the samples are genotyped with Illumina HumanCNV370 or HumanOmniExpress according to the Illumina protocol and the samples were assigned to discovery and replication by the availability on the time of analyses. Data quality control was performed with PLINK (<http://pngu.mgh.harvard.edu/purcell/plink>) (SNP call rate>98%; sample call rate >95%; MAF >0.01; HWE P >10^-6^; cryptic relatedness). Imputation was performed with IMPUTE v1.0 (CEU HapMap rel22 build 36) and association analyses were carried out with SNPTEST.

***Finnish Twin Study on Aging - FITSA***

Finnish Twin Study on Aging (FITSA) (Parssinen et al. 2010) is a study of genetic and environmental effects on the disablement process in older female twins. The FITSA participants were 103 MZ and 114 DZ twin pairs (424 individuals, all Caucasian women) aged 63-76 years living in Finland who took part in multiple laboratory examination in 2000, 2003 and responded in questionnaires in 2011. Before the examinations, the subjects provided a written informed consent according to the Declaration of Helsinki. The study protocol was approved by the ethics committee of the Central Hospital District of Central Finland.

DNA was extracted from EDTA-anticoagulated whole blood according to standard procedures. Because the genotyping was part of a larger project, the GenomEUtwin project, three different genotyping platforms, the MegaBACE1000 (Amersham Biosciences) electrophoresis system, the ABI3700, and the ABI3730 (Applied Biosystems) automated electrophoresis systems were used. The genotype calls were made with the GeneticProfiler1.5 (MegaBACE1000) and GeneMapper3.7 (ABI3700 and ABI3730) software. Oxford Impute-program was used to generate the Hapmap2 imputed SNPs.

***Framingham Eye Study***The Framingham Eye Study (Leibowitz et al. 1980) (FES) was nested within the Framingham Heart Study (FHS, http://www.framinghamheartstudy.org), which began its first round of extensive physical examinations in 1948 by recruiting 5,209 men and women from the town of Framingham, MA, USA. Surviving participants from the original cohort returned for biennial exams, which continue to the present. A total of 2675 FHS participants were also examined as part of the FES between 1973 and 1975. The FES was designed to evaluate ocular characteristics of examinees such as: senile cataract; age-related macular disease; glaucoma; and retinopathy. Between 1989 and 1991, 1603 offspring of original cohort participants also received ocular examinations (1996). The analyses in the current study are limited 1500 (42.5% men) participants from both the original and the offspring cohorts for whom genotype data were available. Most individuals in this analysis set are unrelated but a small number of related pairs remain. Exclusion criteria for refractive error analyses included: visual acuity worse than 20/200; eye absent; aphakia or pseudophakia; and stromal corneal opacity. If any of these conditions were present in only one eye, the other eye was used in the analyses. All data--including refractive error, demographics and genotypes--were retrieved from the database of Genotypes and Phenotypes (dbGaP, http://www.ncbi.nlm.nih.gov/gap) after approval for controlled access to individual-level data. All study protocols are in compliance the World Medical Association Declaration of Helsinki. Since 1971, written consent has been obtained from participants before each examination. The research protocols of the Framingham Heart Study are reviewed annually by the Institutional Review Board of the Boston University Medical Center and by the Observational Studies Monitoring Board of the National Heart, Lung and Blood Institute.
Genotyping was conducted as part of the NHLBI Framingham SNP Health Association Resource (SHARe). This sub-study contains genotype data for approximately 550000 SNPs (Affymetrix 500K mapping arrays [Mapping250k_Nsp and Mapping250K_Sty] plus Affymetrix 50K supplemental human gene-focused array) in over 9200 FHS participants (1500 of whom were used in this analysis). Samples were chosen based on pedigree information and genotyping quality; Samples with a genotypic call rate below 95% were not chosen for analysis. The mean call rate for analyzed samples was 99.2% (SD=0.4%). Genotype data cleaning was carried-out in several steps. The final marker list contained 436494 high-quality SNPs with a minor-allele frequency >= 0.01, a Mendelian error rate below 2% across all pedigrees, a genotype call rate above 95%, and whose distribution was consistent with Hardy-Weinberg expectations (P>0.0001). Genotype imputation to the HapMap-II reference panel (CEU population release 22, NCBI build 36) was carried out in a two-step process using the Markov Chain Haplotyping (MACH version 1.0.16.a) software. First, crossover and error-rate maps were built using 400 unrelated individuals (200 male and 200 female) sampled from FHS subjects. Second, genotype imputations of approximately 2.5 million autosomal HapMap-II SNPs were carried out on the entire FHS dataset using parameters estimated from step 1.

***Gutenberg Health Study (GHS I, GHS II)***

The Gutenberg Health Study (GHS) is a population-based, prospective, observational cohort study in the Rhine-Main Region in midwestern Germany with a total of 15,000 participants and follow-up after five years. The study sample is recruited from subjects aged between 35 and 74 years at the time of the exam. The sample was drawn randomly from local governmental registry offices and stratified by gender, residence (urban and rural) and decade of age. Exclusion criteria were insufficient knowledge of the German language to understand explanations and instructions, and physical or psychic inability to participate in the examinations in the study center. Individuals were invited for a 5-hour baseline-examination to the study center where clinical examinations and collection of blood samples were performed. An important feature of the study design is the interdisciplinary combination of an ophthalmological examination, general and especially cardiovascular examinations, psychosomatic evaluation, laboratory tests, and biobanking for proteomic and genetic analyses. All participants underwent an ophthalmological investigation of 25 minutes’ duration taking place between 11:00 a.m. and 8:00 p.m. This examination was based on standard operating procedures and included a medical history of eye diseases, autorefraction and visual acuity testing (Humphrey^®^ Automated Refractor/Keratometer (HARK) 599™, Carl Zeiss Meditec AG, Jena, Germany), visual field screening using frequency doubling technology (Humphrey^®^ Matrix Perimeter, Carl Zeiss Meditec AG, Jena, Germany), central corneal thickness and keratometry measurement (Scheimpflug imaging with the Pachycam™, Oculus, Wetzlar, Germany), IOP measurement with a non-contact tonometer (Nidek NT-2000™, Nidek Co., Japan), slitlamp biomicroscopy with undilated pupils (Haag-Streit BM 900^®^, Bern, Switzerland) and non-mydriatic fundus photography (Visucam PRO NM,™, Carl Zeiss Meditec AG, Jena, Germany), all administered by an ophthalmologist. The study was approved by the Medical Ethics Committee of the University Medical Center Mainz and by the local and federal data safety commissioners. According to the tenets of the Declaration of Helsinki, written informed consent was obtained from all participants prior to entering the study.
Within GHS, DNA was extracted from buffy-coats from EDTA blood samples as described in Zeller *et al*. (Zeller et al. 2010). Genetic analysis was conducted in the first 5,000 study participants. For these, 3,463 individuals were genotyped in 2008 (GHS I) and further 1,439 individuals in 2009 (GHS II). Genotyping was performed for GHS I and GHS II using the Affymetrix Genome-Wide Human SNP Array 6.0 (<http://www>.affymetrix.com), as described by the Affymetrix user manual. Genotypes were called using the Affymetrix Birdseed-V2 calling algorithm. Individuals with a call rate below 97% or a too high autosomal heterozygosity (3 s.d. from mean) and sex-mismatches were excluded. After applying standard quality criteria (minor allele frequency >1%, genotype call rate >98% and P-value of deviation from Hardy-Weinberg equilibrium of >0.0001), 675,350 SNPs in 2,996 individuals from GHS I and 673,914 SNPs in 1,179 individuals from GHS II remained for analysis. Imputation of missing genotypes was performed using Impute software v2.1.0 and HapMap release 24, NCBI Build 36.

***KORA***KORA ("Kooperative Gesundheitsforschung in der Region Augsburg" which translates as “Cooperative Health Research in the Region of Augsburg”) is a population based study of adults randomly selected from 430,000 inhabitants living in Augsburg and 16 surrounding counties in Germany (Holle et al. 2005; Oexle et al. 2011; Steffens et al. 2006; Wichmann et al. 2005). The collection was done in 4 separate groups from 1984-2001 (S1-S4). All survey participants are residents of German nationality identified through the registration office. In the KORA S3 and S4 studies 4,856 and 4,261 subjects have been examined implying response rates of 75% and 67%, respectively. 3,006 subjects participated in a 10-year follow-up examination of S3 in 2004/05 (KORA F3), and 3080 of S4 in 2006/2008 (KORA F4). The age range of the participants was 25 to 74 years at recruitment. The study was approved by the local ethics committee. Written informed consent was obtained from all participants before enrollment in accordance with the Declaration of Helsinki. A genome-wide association study of refractive error using the Illumina 2.5M chip was performed on a subset of 1981 individuals in the S3/F3 (mean age 55.7, range 35–84) who had measurements of refractive error and available DNA samples. For each subject, eyeglass prescriptions were measured in addition to an evaluation using the Nikon Retinomax. The individuals included in this GWAS are all Caucasian, do not have age-related macular degeneration, cataracts, retinitis pigmentosa, color blindness, other congenital eye problems, LASIK, artificial lenses, and other eye surgery. Refractive error was analyzed, taking the mean measured spherical equivalent (SE) across both eyes (or SE in a single eye when both eyes were not measured) as the trait of interest. Age and gender were also included as covariates. DNA was extracted from cell lines according to standard protocols. Genotyping of SNPs was performed using the Illumina HumanOmni2.5-4v1_B chip array. ). Samples with low call rate (<98%), with low mean confidence scores over all non-missing genotypes, with chromosome anomalies, or with sex-mismatch were excluded. No samples exhibited excess heterozygosity rates (1.5 interquartile ranges above or below the upper/lower quartile ranges). Cryptic relatedness was detected by estimating IBD sharing and kinship coefficients among all possible pairs and one member of each pair that exhibited a sibling or closer relationship was dropped from the analysis. SNPs were dropped from the analysis if they exhibited more than 1 blind duplicate error, more than 1 HapMap control error or more than 1 error in HapMap control trios, a genotype call rate < 99%, minor allele frequency < 0.01, or Hardy-Weinberg *P*-value < 10^−4^. Tests for batch effects were not significant. No sex-specific differences in allelic frequency (>0.2) or heterozygosity (>0.3) were detected. Eigenstrat did not detect significant population stratification and the genomic control inflation factor was 1.014. A subset of the retained SNPs was used for imputation with the Markov Chain Haplotyping (MACH) package version 1.0.17 software (imputed to plus strand of NCBI build 36, HapMap release #22). For each imputed SNP, a reliability of imputation was estimated as the ratio of the empirically observed dosage variance to the expected binomial dosage variance (O/E ratio). PLINK (<http://pngu.mgh.harvard.edu/purcell/plink>) was used to perform the association analyses.

***Kyoto Study***Japanese pathological myopic cases were recruited at the Center for Macular Diseases of Kyoto University Hospital, the High Myopia Clinic of Tokyo Medical and Dental University, and Fukushima Medical University Hospital. All subjects underwent comprehensive ophthalmologic examinations, including dilated indirect and contact lens slit-lamp biomicroscopy, automatic objective refraction evaluation, and measurement of the axial length by applanation A-scan ultrasonography or partial coherence interferometry. As a general population control, 3120 Japanese individuals were recruited at Kyoto University Hospital and Aichi Cancer Center Research Institute. We also used DNA samples from 929 subjects who were randomly selected from the Pharma SNP Consortium (PSC); this group has been used for previous genomic studies and is regarded as being representative of the general Japanese population. All procedures used in this study conformed to the tenets of the Declaration of Helsinki. The Institutional Review Board and the Ethics Committee of each institution approved the protocols used. All the participants were fully informed of the purpose and procedures, and a written consent was obtained from each.
Genomic DNAs were extracted from peripheral blood leukocytes with QuickGene-610L DNA extraction kit (FUJIFILM Co., Tokyo, Japan). In 483 cases and 2899 controls, Genotyping of SNPs was performed using the Illumina HumanHap550 or HumanHap610 chips (Illumina Inc., San Diego, CA). A systematic quality control procedure of the genome scan results was applied as follows. Samples were evaluated for data quality first and markers were subsequently excluded. Genetic proximity of sample pairs was evaluated with pi-hat in PLINK (<http://pngu.mgh.harvard.edu/purcell/plink>) and samples with indication of kinship or sample duplication were excluded. Genotypes in X chromosome were used for checking the precision of the phenotype record, and samples with mismatch in gender were removed. We excluded SNPs with low successful call rate (<95%), with the distortion of Hardy-Weinberg Equilibrium (P<10−3 by HWE exact test), and with minor allele frequency less than 0.05. In 1140 cases and 929 PSC controls, Genotyping of SNPs was performed using the Taqman SNP assay with the ABI PRISM 7700 system (Applied Biosystems, Foster City, CA). Of the 483 cases genotyped with Illumina Infinium assay, 480 samples were also genotyped with Taqman assay.

***MESA***The Multi-Ethnic Study of Atherosclerosis (MESA) is a study of the characteristics of subclinical cardiovascular disease (disease detected non-invasively before it has produced clinical signs and symptoms) and the risk factors that predict progression to clinically overt cardiovascular disease or progression of the subclinical disease. MESA researchers study a diverse, population-based sample of 6,814 asymptomatic men and women aged 45-84. Thirty-eight percent of the recruited participants are white, 28 percent African-American, 22 percent Hispanic, and 12 percent Asian, predominantly of Chinese descent (Bild et al. 2002). Participants were recruited from six field centers across the United States. Phenotype (spherical equivalent) and genotype data were available for 1462 Caucasian subjects. The tenets of the Declaration of Helsinki were followed and institutional review board approval was granted at all MESA sites. Written informed consent was obtained from each participant.
Genotyping was performed using the Affymetrix Genome-Wide Human SNP Array 6.0. IMPUTE version 2.1.0 was used to perform imputation for the MESA Caucasian participants (chromosomes 1-22) using HapMap Phase I and II - CEU as the reference panel (release #24 - NCBI Build 36 (dbSNP b126)). SNPs with MAF less than 0.02 or HWE p value less than 0.001 were removed from the analysis. Association tests were performed by SNPTEST v2 (Marchini et al. 2007).

***Orkney Complex Disease Study (ORCADES)***

The Orkney Complex Disease Study (ORCADES) is a population-based, cross-sectional study in the Scottish archipelago of Orkney, including 1,285 individuals with eye measurements. The study received approval from relevant ethics committees in Scotland and followed the tenets of the Declaration of Helsinki. Autorefractive measurements were obtained using a Kowa KW 2000 autorefractometer. Measures on eyes with a history of trauma, intra-ocular surgery or LASIK operations were removed and the analysis was done on the right eye measures, unless the left eye had more complete measurements (e.g. due to trauma or cataract surgery on the right eye) (Vitart et al. 2010b). Extreme values (lying more than 3 interquartile range from the upper or lower quartile) were removed. 505 individuals which had been genotyped and passed genotyping quality control were used in this analysis. Inverse normal transformed spherical equivalent refraction adjusted for age and age squared was used as outcome in the genetic association analysis and obtained using the rank transformation function in GenABEL (http://www.genabel.org/). Genome-wide association analysis was performed using the ProbABEL package using an additive SNP allelic effect model and correcting for individual relatedness using the polygenic and mmscore functions implemented in the GenABEL package.

***Rotterdam Study (RS 1, RS 2, RS 3)***

The Rotterdam Study is a prospective population-based cohort study in the elderly living in Ommoord, a suburb of Rotterdam, the Netherlands. Details of the study are described elsewhere (Hofman et al. 2011). In brief, the Rotterdam Study consists of 3 independent cohorts: RS 1, RS 2, and RS 3. For the current analysis, 5,328 residents aged 55 years and older were included from RS 1, 2,009 participants aged 55 and older from RS 2, and 1,970 aged 45 and older from RS 3. 99% of subjects were of Caucasian ancestry. Participants underwent multiple physical examinations with regular intervals from 1991 to present, including a non-dilated automated measurement of refractive error using a Topcon RM-A2000 autorefractor. All measurements in RS-I–III were conducted after the Medical Ethics Committee of the Erasmus University had approved the study protocols and all participants had given a written informed consent in accordance with the Declaration of Helsinki.
DNA was extracted from blood leucocytes according to standard procedures. Genotyping of SNPs was performed using the Illumina Infinium II HumanHap550 chip v3.0 array (RS-I); the HumanHap550 Duo Arrays and the Illumina Human610-Quad Arrays (RS-II), and the Human 610 Quad Arrays Illumina (RS-III). Samples with low call rate (<97.5%), with excess autosomal heterozygosity (>0.336), or with sex-mismatch were excluded, as were outliers identified by the identity-by-state clustering analysis (outliers were defined as being >3 s.d. from population mean or having identity-by-state probabilities >97%). GWAS analyses were performed using GRIMP. We used genomic control to obtain optimal and unbiased results and applied the inverse variance method of each effect size estimated for both autosomal SNPs that were genotyped and imputed in both cohorts. A set of genotyped input SNPs with call rate >98%, with minor allele frequency >0.01, and with Hardy-Weinberg P value >10^−6^ was used for imputation. We used the Markov Chain Haplotyping (MACH) package version 1.0.15 software (Rotterdam, The Netherlands; imputed to plus strand of NCBI build 36, HapMap release #22) for the analyses. For each imputed SNP, a reliability of imputation was estimated as the ratio of the empirically observed dosage variance to the expected binomial dosage variance (O/E ratio).

***OGP Ogliastra Genetic Park - Talana study (OGP Talana)***A cross-sectional ophthalmic study was performed in Talana, Perdasdefogu and Urzulei within the Ogliastra Project, a large epidemiological survey conducted in a geographically, culturally and genetically isolated population living in an eastern-central region of Sardinia (Biino et al. 2005). In Talana the study was carried out between October 2001 and October 2002 and adhered to the tenets of the declaration of Helsinki. Talana is an Ogliastran village situated at an altitude of 700 m above sea level in one of the most secluded areas of Sardinia; it has about 1200 inhabitants and, importantly, archival records are available from 1589 and genealogical trees have been reconstructed from 1640. 789 volunteers gave their written informed consent and were invited to the local medical centre, which was equipped with a complete set of ophthalmic instruments for this survey. All participants underwent a complete eye examination conducted according to a standardized protocol that included visual acuity measurement with Snellen charts at a distance of 5 m, autorefraction (RK-8100 Topcon, Tokyo, Japan) assessing sphere, cylinder and axis, slit lamp biomicroscopy (Model BQ900, Haag-Streit, Bern, Switzerland), contact tonometry and colour fundus photography (TRC-50IA,Topcon) and non-contact optical biometry (IOLMaster,Carl Zeiss, Italy) and Optical coherence tomography (OCT). Whole blood was obtained from all consenting family members of Talana village for DNA extraction. Genotyping was carried out using the Affymetrix 500k chips using standard protocols. SNPs quality control was performed using the GenABEL software package in R. Samples with overall SNP call rate < 93%, with minor allele frequency < 0.01, and with Hardy-Weinberg P value >10^−6^, showing excess of heterozigosity, or being classified as outliers by allelic identity-by-state (IBS) clustering analysis, were excluded. Using the phase II CEU HapMap individuals (release 22, NCBI build 36) as reference panel for imputation, we imputed genotypes to nearly 2.5 milion SNPs using MACH. SNPs imputed with Rsq <0.3 were excluded. All regression models were run using the ProbABEL package from the ABEL set of programs.

***SORBS***All subjects are part of a sample from an extensively phenotyped self-contained population from Eastern Germany, the Sorbs (Tonjes et al. 2009). At present, about 1000 Sorbian individuals are enrolled in the study. Extensive phenotyping included standardised questionnaires for past medical history and family history, collection of anthropometric data and a 75g-glucose-tolerance-test. Genotyping was performed using 500K Affymetrix GeneChip (Affymetrix, Inc) and Affymetrix Genome-Wide Human SNP Array 6.0. QC filters for genotyped SNPs used for imputation were: MAF<1%, pHWE<10^-4^, call rate<95%. 378513 SNPs were used for imputation (Impute, HapMap CEU release 21 (build 35)).
The study was approved by the ethics committee of the University of Leipzig and all subjects gave written informed consent before taking part in the study.

***TwinsUK***The TwinsUK adult twin registry based at St. Thomas’ Hospital in London is a volunteer cohort of over 10,000 twins from the general population (Spector and Williams 2006). Twins largely volunteered unaware of the eye studies, gave fully informed consent under a protocol reviewed by the St. Thomas’ Hospital Local Research Ethics Committee and underwent non-cyclopleged autorefraction using an ARM-10 autorefractor (Takagi Ltd). Out of the original 4,388 subjects for whom phenotype and genotype information was available, 4,270 subjects were included in this study; 118 subjects were excluded after failing quality control.
Genotyping was carried out using three genotyping platforms from Illumina: the HumanHap 300k Duo for part of the UK Twin Cohort and the HumanHap610-Quad array for the rest of the UK Twin Cohort. Imputation was calculated with reference to HapMap release 22 CEU population data using IMPUTE version 2. Individuals were included if their genotyping success rate exceeded 95%,did not show excess or low heterozygosity (defined by the interval interval of 0.2-04). SNPs were included in the imputation if they had a genotype success rate of at least 0.95 if their minor allele frequency was superior to 0.005 and at least 0.99 if their MAF was 0.01-0.05. Only SNPs that were within Hardy-Weinberg equilibrium (p>10^-04^) and had a minor allele frequency of 0.04 or above were regressed.

***Young Finns Study (YFS)***

**The YFS cohort is a Finnish longitudinal population study sample on the evolution of cardiovascular risk factors from childhood to adulthood (Raitakari et al. 2008). The first cross-sectional study was conducted in the year 1980 in five different centers. It included 3,596 participants in the age groups of 3, 6, 9, 12, 15, and 18, who were randomly chosen from the national population register. After the baseline in 1980 these subjects have been re-examined in 1983 and 1986 as young individuals, and in 2001, 2007 and 2011 as older individuals. For the current analysis a subsample from the newest (2011) follow-up was used from Tampere and Turku (N=397, aged 33-48) where the refractive error measurements data from both eyes were available.** This study was carried out in accordance with the recommendations of the Declaration of Helsinki. All participants provided written informed consent and the study protocol was approved by the Ethics Committee.
Genomic DNA was extracted from peripheral blood leukocytes using a commercially available kit and Qiagen BioRobot M48 Workstation according to the manufacturer’s instructions (Qiagen, Hilden, Germany). Genotyping was done for 2,556 samples using custom build Illumina Human 670k BeadChip at Welcome Trust Sanger Institute. Genotypes were called using Illuminus clustering algorithm. 56 samples failed Sanger genotyping pipeline QC criteria (i.e., duplicated samples, heterozygosity, low call rate, or Sequenom fingerprint discrepancy). From the remaining 2,500 samples one sample failed gender check, three was removed due to low genotyping call rate (< 0.95) and 54 samples for possible relatedness (pi-hat > 0.2) . 11,766 SNPs were excluded based on Hardy–Weinberg equilibrium (HWE) test (p ≤ 10^-6^), 7,746 SNPs failed missingness test (call rate < 0.95 ) and 34,596 SNPs failed frequency test (MAF < 0.01). After quality control there were 2,442 samples and 546,677 genotyped SNPs available for further analysis (Smith et al. 2010). Genotype imputation was performed using MACH (Li et al. 2009; Li et al. 2010) 1.0 and HapMap II CEU (release 22, NCBI build 36, dbSNP 126) samples as reference. Palindromic A/T and C/G SNPs were removed before imputation. After imputation there were 2,543,887 SNPs available. SNPs with squared correlation between imputed and true genotypes ≥ 0.30 were considered well imputed.

***Singapore cohorts - SCORM, SP2, SIMES SINDI***

*Singapore Cohort study of the Risk factors for Myopia (SCORM)*
A total of 1,979 children in grades 1, 2, and 3 from three schools were recruited from 1999 to 2001. The children were examined on the school premises every year by a team of eye care professionals (Saw et al. 2006). The GWA study was conducted in a subset of Chinese children of 1,116 subjects, comprising 56% of the whole cohort (Fan et al. 2011; Li et al. 2011). The phenotype used in this study was based on the Spherical Equivalent (SE) obtained on the 4^th^ annual examination of the study (children at age 10 to 12 years).
A total of 1116 DNA samples (1037 from buccal swab and 79 from saliva) were genotyped on the Illumina HumanHap 550 Beadchips or 550 Duo Beadarrays. Of them, 187 samples were excluded, including: (i) 70 samples with call rates below 98%; (ii) 6 with poor genotyping quality; (iii) 11 sib-ships; (iv) 18 with inconsistent gender information, v) 3 due to population structure. This left a total of 1,008 samples for further SNP QC. Based on 514,849 autosomal SNPs, we excluded 31,457 markers if they had missing genotype calls > 10%, a minor allele frequency < 1%, or significantly deviated from HWE (p < 10^-6^).

*Singapore Prospective Study Program (SP2)*
Samples of SP2 were from a revisit of two previously conducted population-based surveys carried out in Singapore between 1992 and 1998, including the National Health Survey 1992 and the National Health Survey 1998 (Hughes et al. 1997). These studies comprise random samplings of individuals stratified by ethnicity from the entire Singapore population. A total of 8266 subjects were invited in this follow-up survey and 6301 (76.1% response rate) subjects completed the questionnaire, of which 4056 (64.4% of those who completed the questionnaire) also attended the health examination and donated blood specimens. The present GWA genotyping for SP2 involved individuals of Chinese descent only (n=2,867) (Sim et al. 2011).
Of the 2,867 blood-derived DNA samples, 392 samples were genotyped on the HumanHap 550v3, 1,459 samples on the 610-Quad, 817 samples on the 1M-Duov3, 191 samples on both 550v3 and 1M-Duov3, and 8 samples on both 610-Quad and 1M-Duov3. For the samples that were genotyped on two platforms, we used the genotypes from the denser platform in our study. We excluded 443 individuals on the following conditions, sample call rates of less than 95%, excessive heterozygosity, cryptic relatedness by IBS, population structure ascertainment, and gender discrepancies as listed in the main text. This left 2,434 post-QC SP2 samples. During the SNPs QC procedure, we excluded SNPs with low genotyping call rates (> 5% missingness) or monomorphic, with MAF < 1%, or with significant deviation from HWE (P< 10^-6^). This yielded a post-QC set of 462,580 SNPs. As SP2 samples are genotyped on different platforms, the concordance of the duplicate samples plated on different Beadarrays chips was also examined as quality of genotyping. The average SNP concordance rate between chips for the post-QC duplicated samples was 0.995. We additionally assessed the SNPs that are present on different platforms for extreme variations in allele frequencies with a 2-degree of freedom chi-square test of proportions, removing 62 SNPs with *P*-values < 0.0001.

*Singapore Malay Eye Study (SiMES*)
SiMES is a population-based prevalence survey of Malay adults aged 40 to 79 years living in Singapore that was conducted between August of 2004 and June of 2006 (Foong et al. 2007). From a Ministry of Home Affairs random sample of 16,069 Malay adults in the Southwestern area, an age-stratified random sampling strategy was used in selecting 1400 from each decade from age 40 years onward (40–49, 50–59, 60–69, and 70–79 years).The 4,168 eligible participants from the sampling frame, while 3280 (78.7%) participated. Genome-wide genotyping was performed in 3,072 individuals (Cornes et al. 2012; Vithana et al. 2011).
Total of 3,072 DNA samples were genotyped using the Illumina Human 610 Quad Beadchips (Khor et al. 2011; Vithana et al. 2011). Using the same quality control criteria, we omitted a total of 530 individuals including those of subpopulation structure (n=170), cryptic relatedness (n=279), excessive heterozygosity or high missingness rate > 5% (n=37), and gender discrepancy (n=44). After the removal of the samples, SNP QC was then applied on a total of 579,999 autosomal SNPs for the 2,542 post-QC samples. SNPs were excluded based on (i) high rates of missingness (> 5%) ; (ii) monomorphism or MAF < 1% ; or (iii) genotype frequencies deviated from HWE (p <1 × 10^-6^).

*Singapore Indian Eye Study (SINDI)*
SINDI is a population-based survey of major eye diseases (Lavanya et al. 2009) in ethnic Indians aged 40 to 80 years living in the South-Western part of Singapore and was conducted from August 2007 to December 2009. In brief, 4,497 Indian adults were eligible and 3,400 participated. Genome-wide genotyping was performed in 2,953 individuals (Khor et al. 2011). As in the discovery cohorts, participants were excluded from the study if they had cataract surgery and missing refraction data.
The Illumina Human610 Quad Beadchips was used for genotyping all DNA samples from SINDI (n=2,593). We excluded 415 subjects from the total of 2,953 genotyped samples based on: excessive heterozygosity or high missingness rate > 5% (n=34) , cryptic relatedness (n=326), issues with population structure ascertainment (n=39) and gender discrepancies (n=16). This left a total of 2,538 individuals with 579,999 autosomal SNPs. During SNP QC procedure. SNPs were excluded based on (i) high rates of missingness (> 5%) ; (ii) monomorphism or MAF < 1% ; or (iii) genotype frequencies deviated from HWE (p <1 × 10^-6^).

All four studies adhere to the Declaration of Helsinki. Ethics approvals have been obtained from the Institutional Review Boards of the Singapore Eye Research Institute, Singapore General hospital, National University of Singapore and National Healthcare Group, Singapore. In all cohorts, participants provided written, informed consent at the recruitment into the studies. For studies involving children (SCORM), written informed consent was obtained from the children’s parents.

**Supplementary Table 1** Phenotyping and genotyping methods for all study cohorts

| **Study** | **Measurement of refractive error** | **GWAS chip** | **Imputation** |
| --- | --- | --- | --- |
| 1958 British Birth Cohort | Nikon Retinomax 2 | Illumina’s Human1M-Duo chip | Mach1 |
| AGES Reykjavik | NIDEK ARK760A | Illumina Human 370CNV-Duo | Hapmap CEU, build 36, release 22, MACH v1.0.16 |
| ALSPAC | Canon R50 model autorefractor | Illumina HumanHap550 quad | Hapmap CEU build 36, release 22, MACH v1.0.15 |
| AREDS 1 | Subjective Refraction | Affymetrix 100K, Illumina 100K, Illumina 300K | HapMap II |
| AREDS 2 | Subjective Refraction | Illumina 2.5M | Hapmap CEU, build 36, release 22, hg19, MACH v1.0.17 |
| Australian Twins | Humphrey-598 automatic refractor (Carl Zeiss Meditec, Inc.) | Illumina HumanHap610-Quad | Mach with Merlin |
| BMES | Humphrey-530 automatic refractor (Allergan Humphrey) | Illumina Human 670-Quad | MACH v 1.1.16 |
| Croatia Split | NIDEK ARK30 | 370CNV-Quadv3 | Hapmap CEU, build 36, release 22, MACH v1.0.15 |
| Croatia Vis Island | NIDEK ARK30 | HumanHap 300v1 | Hapmap CEU, build 36, release 22, MACH v1.0.15 |
| Croatia Korcula Island | NIDEK ARK30 | 370CNV-Quad | Hapmap CEU, build 36, release 22, MACH v1.0.15 |
| ERF | Topcon RM-A2000 autorefractor | Illumina 6k, Illumina 318K, Illumina 370K and Affymetrix 250K | Hapmap CEU, build 36, release 22, MACH v1.0.15 |
| EGCUT | Topcon KR 7000S and eyeglass prescriptions | Illumina Human370CNV and OmniExpress | Impute v1.0, build 36.3 and rel22 |
| Finnish Twin Study on Aging | Topcon AT, Tokyo, Japan | Illumina 300 K | Hapmap2, Impute |
| Framingham Eye Study | Manifest refraction | Affy GeneChip Human Mapping 250K Nsp ArrayAffy GeneChip Human Mapping 250K Sty Array, AFFY HuGene Focused 50K array | Hapmap 3 using MACH 3 |
| Gutenberg Health Study I | Humphrey Automated Refractor 599 (Zeiss) | Affymetrix Genome-Wide Human SNP 6.0 Array | Impute v2.1.0 (HapMap-build b36 r24) |
| Gutenberg Health Study II | Humphrey Automated Refractor 599 (Zeiss) | Affymetrix Genome-Wide Human SNP 6.0 Array | Impute v2.1.0 (HapMap-build b36 r24) |
| KORA | Nikon Retinomax and eyeglass prescriptions | HumanOmni2.5-4v1_B | Hapmap CEU, build 36, release 22, hg19, MACH v1.0.17 |
| MESA | NIDEK ARK-760A | Affymetrix GeneChip SNP Array 6.0. | Hapmap CEU, build 36, release 24, IMPUTE 2.1.0 |
| ORCADES | Kowa KW 2000 | HumanHap 300v2 and 370CNV-Quad | Hapmap CEU, build 36, release 22, MACH v1.0.15 |
| Rotterdam Study 1 | Topcon RM-A2000 autorefractor | Illumina Infinium II HumanHap550 chip v3.0 array | Hapmap CEU, build 36, release 22, MACH v1.0.15 |
| Rotterdam Study 2 | Topcon RM-A2000 autorefractor | HumanHap550 Duo Arrays + Human610-Quad Arrays Illumina | Hapmap CEU, build 36, release 22, MACH v1.0.15 |
| Rotterdam Study 3 | Topcon RM-A2000 autorefractor | Human 610 Quad Arrays Illumina | Hapmap CEU, build 36, release 22, MACH v1.0.15 |
| OGP Talana | Autorefractor RK-8100 Topcon, Tokyo, Japan | Affymetrix 500K | Hapmap CEU, build 36, release 22, MACH v1.0.15 |
| SCORM | Canno RK-5 autorefractor | Illumina HumanHap 550 / 550 Duo Beadarrays | HapMap CHB+JPT, build 36, release 22, IMPUTEv0.5.0 |
| SiMES | Canno RK-5 autorefractor | Illumina 610Quad Chip | HapMap combined panel of all four populations, build 36, release 22, IMPUTEv0.5.0 |
| SINDI | Canno RK-5 autorefractor | Illumina 610Quad Chip | HapMap combined panel of all four populations, build 36, release 22, IMPUTEv0.5.0 SP2:HapMap CHB+JPT, build 36, release 22, IMPUTEv0.5.0 |
| SP2 | Canno RK-5 autorefractor | Illumina HumanHap 550v3 / 610Quad / 1M-Duov3 | HapMap CHB+JPT, build 36, release 22, IMPUTEv0.5.0 |
| TwinsUK | ARM-10 autorefractor (Takagi Ltd) | HumanHap 300k Duo and HumanHap610-Quad array | Impute2 |
| Young Finns | Nidek, AR-310A | Illumina 670K Custom Array | Hapmap CEU, build 36, release 22, MACH v1.0 |
| Kyoto Study | NIDEK ARK-530A | Illumina HumanHap 550/Taqman | Hapmap JPT, build 36, release 22, MACH v1.0.15 |
| SORBS | History of myopia in standardized interview | Affymetrix 500k and Affymetrix 6.0 | HapMap2 NCBI build 35 |

**Supplementary Table 2** Exact sample size of each SNP per study

|  | **Total** | **1958BBC** | **AGES** | **ALSPAC** | **AREDS 1** | **AREDS 2** | **Australian Twins** | **BMES** | **Croatia Split** | **Croatia Vis Island** | **Croatia Korcula Island** | **ERF** | **EGCUT** | **FITSA** | **Framingham Eye Study** | **GHS I** | **GHS II** | **KORA** | **MESA** | **ORCADES** | **RS 1** | **RS 2** | **RS 3** | **OGP Talana** | **SCORM** | **SiMES** | **SINDI** | **SP2** | **TwinsUK** | **Young Finns** |
| --- | --- | --- | --- | --- | --- | --- | --- | --- | --- | --- | --- | --- | --- | --- | --- | --- | --- | --- | --- | --- | --- | --- | --- | --- | --- | --- | --- | --- | --- | --- |
| locus 15q14 |  |  |  |  |  |  |  |  |  |  |  |  |  |  |  |  |  |  |  |  |  |  |  |  |  |  |  |  |  |  |
| rs634990 | **49056** | 1658 | 2986 | 3804 | 816 | 1506 | 1819 | 1574 | 366 | 544 | 836 | 2032 | 158 | 0 | 1500 | 2745 | 1142 | 1867 | 1461 | 505 | 5328 | 2009 | 1970 | 623 | 929 | 2226 | 2055 | 1930 | 4270 | 397 |
| rs560766 | **48974** | 1658 | 2986 | 3804 | 816 | 1506 | 1819 | 1574 | 366 | 544 | 836 | 2032 | 75 | 0 | 1500 | 2745 | 1142 | 1867 | 1462 | 505 | 5328 | 2009 | 1970 | 623 | 929 | 2226 | 2055 | 1930 | 4270 | 397 |
| rs524952 | **49201** | 1658 | 2986 | 3804 | 816 | 1506 | 1819 | 1574 | 366 | 544 | 836 | 2032 | 176 | 127 | 1500 | 2745 | 1142 | 1867 | 1461 | 505 | 5328 | 2009 | 1970 | 623 | 929 | 2226 | 2055 | 1930 | 4270 | 397 |
| rs688220 | **48935** | 1658 | 2986 | 3804 | 816 | 1506 | 1819 | 1574 | 366 | 544 | 836 | 2032 | 36 | 0 | 1500 | 2745 | 1142 | 1867 | 1462 | 505 | 5328 | 2009 | 1970 | 623 | 929 | 2226 | 2055 | 1930 | 4270 | 397 |
| rs580839 | **48966** | 1658 | 2986 | 3804 | 816 | 1506 | 1819 | 1574 | 366 | 544 | 836 | 2032 | 67 | 0 | 1500 | 2745 | 1142 | 1867 | 1462 | 505 | 5328 | 2009 | 1970 | 623 | 929 | 2226 | 2055 | 1930 | 4270 | 397 |
| rs11073060 | **48961** | 1658 | 2986 | 3804 | 816 | 1506 | 1819 | 1574 | 366 | 544 | 836 | 2032 | 62 | 0 | 1500 | 2745 | 1142 | 1867 | 1462 | 505 | 5328 | 2009 | 1970 | 623 | 929 | 2226 | 2055 | 1930 | 4270 | 397 |
| rs4924134 | **48962** | 1658 | 2986 | 3804 | 816 | 1506 | 1819 | 1574 | 366 | 544 | 836 | 2032 | 64 | 0 | 1500 | 2745 | 1142 | 1867 | 1461 | 505 | 5328 | 2009 | 1970 | 623 | 929 | 2226 | 2055 | 1930 | 4270 | 397 |
| rs7176510 | **48947** | 1658 | 2986 | 3804 | 816 | 1506 | 1819 | 1574 | 366 | 544 | 836 | 2032 | 49 | 0 | 1500 | 2745 | 1142 | 1867 | 1461 | 505 | 5328 | 2009 | 1970 | 623 | 929 | 2226 | 2055 | 1930 | 4270 | 397 |
| rs619788 | **48964** | 1658 | 2986 | 3804 | 816 | 1506 | 1819 | 1574 | 366 | 544 | 836 | 2032 | 66 | 0 | 1500 | 2745 | 1142 | 1867 | 1461 | 505 | 5328 | 2009 | 1970 | 623 | 929 | 2226 | 2055 | 1930 | 4270 | 397 |
| rs7163001 | **48961** | 1658 | 2986 | 3804 | 816 | 1506 | 1819 | 1574 | 366 | 544 | 836 | 2032 | 63 | 0 | 1500 | 2745 | 1142 | 1867 | 1461 | 505 | 5328 | 2009 | 1970 | 623 | 929 | 2226 | 2055 | 1930 | 4270 | 397 |
| rs11073059 | **48960** | 1658 | 2986 | 3804 | 816 | 1506 | 1819 | 1574 | 366 | 544 | 836 | 2032 | 62 | 0 | 1500 | 2745 | 1142 | 1867 | 1461 | 505 | 5328 | 2009 | 1970 | 623 | 929 | 2226 | 2055 | 1930 | 4270 | 397 |
| rs11073058 | **48960** | 1658 | 2986 | 3804 | 816 | 1506 | 1819 | 1574 | 366 | 544 | 836 | 2032 | 62 | 0 | 1500 | 2745 | 1142 | 1867 | 1461 | 505 | 5328 | 2009 | 1970 | 623 | 929 | 2226 | 2055 | 1930 | 4270 | 397 |
| rs685352 | **47501** | 0 | 2986 | 3804 | 816 | 1506 | 1819 | 1574 | 366 | 544 | 836 | 2032 | 261 | 0 | 1500 | 2745 | 1142 | 1867 | 1461 | 505 | 5328 | 2009 | 1970 | 623 | 929 | 2226 | 2055 | 1930 | 4270 | 397 |
| rs8032019 | **48968** | 1658 | 2986 | 3804 | 816 | 1506 | 1819 | 1574 | 366 | 544 | 836 | 2032 | 70 | 0 | 1500 | 2745 | 1142 | 1867 | 1461 | 505 | 5328 | 2009 | 1970 | 623 | 929 | 2226 | 2055 | 1930 | 4270 | 397 |
| locus 15q25 |  |  |  |  |  |  |  |  |  |  |  |  |  |  |  |  |  |  |  |  |  |  |  |  |  |  |  |  |  |  |
| rs939661 | **49360** | 1658 | 2986 | 3804 | 816 | 1506 | 1819 | 1574 | 366 | 544 | 836 | 2032 | 338 | 127 | 1500 | 2745 | 1142 | 1864 | 1461 | 505 | 5328 | 2009 | 1970 | 623 | 929 | 2226 | 2055 | 1930 | 4270 | 397 |
| rs939658 | **49350** | 1658 | 2986 | 3804 | 816 | 1506 | 1819 | 1574 | 366 | 544 | 836 | 2032 | 325 | 127 | 1500 | 2745 | 1142 | 1867 | 1461 | 505 | 5328 | 2009 | 1970 | 623 | 929 | 2226 | 2055 | 1930 | 4270 | 397 |
| rs17175798 | **49363** | 1658 | 2986 | 3804 | 816 | 1506 | 1819 | 1574 | 366 | 544 | 836 | 2032 | 338 | 127 | 1500 | 2745 | 1142 | 1867 | 1461 | 505 | 5328 | 2009 | 1970 | 623 | 929 | 2226 | 2055 | 1930 | 4270 | 397 |
| rs8033963 | **49364** | 1658 | 2986 | 3804 | 816 | 1506 | 1819 | 1574 | 366 | 544 | 836 | 2032 | 338 | 127 | 1500 | 2745 | 1142 | 1867 | 1462 | 505 | 5328 | 2009 | 1970 | 623 | 929 | 2226 | 2055 | 1930 | 4270 | 397 |
| rs8027411 | **49360** | 1658 | 2986 | 3804 | 816 | 1506 | 1819 | 1574 | 366 | 544 | 836 | 2032 | 338 | 127 | 1500 | 2745 | 1142 | 1865 | 1460 | 505 | 5328 | 2009 | 1970 | 623 | 929 | 2226 | 2055 | 1930 | 4270 | 397 |

**Supplementary Table 3** Results of the quantitative analysis of 26 additional SNPs at locus 15q25

1 TwinsUK, RS1, RS2, RS3, ERF, 1958 Birth Cohort, Australian Twins (adult samples only)
2 AGES, AREDS 2, BMES, Croatia Split, Croatia Vis, Croatia Korcula, FITSA, Framingham, GHS I, GHS II, KORA, MESA, ORCADES, OGP Talana, SCORM, SiMES, SINDI, SP2, Young Finns
3 AGES, AREDS 2, BMES, Croatia Split, Croatia Vis, Croatia Korcula, FITSA, Framingham, GHS I, GHS II, KORA, MESA, ORCADES, OGP Talana, Young Finns
4 SP2, SIMES, SINDI, SCORM
5 all studies

For these analyses, a Bonferroni corrected *P*-value of 1.92 x 10^-3^ was considered significant (for GWAS data from discovery phase P < 5 × 10^−8^ was considered genome-wide significant).* Freq = average frequency

**Supplementary Figure 1** Distribution of minor allele frequencies at locus 15q25 and 15q14

**References**

(1996) Familial aggregation and prevalence of myopia in the Framingham Offspring Eye Study. The Framingham Offspring Eye Study Group. Arch Ophthalmol 114: 326-32

Age-Related Eye Disease Study Research G (1999) The Age-Related Eye Disease Study (AREDS): design implications. AREDS report no. 1. Control Clin Trials 20: 573-600

Age-Related Eye Disease Study Research G (2001a) A randomized, placebo-controlled, clinical trial of high-dose supplementation with vitamins C and E and beta carotene for age-related cataract and vision loss: AREDS report no. 9. Arch Ophthalmol 119: 1439-52

Age-Related Eye Disease Study Research G (2001b) A randomized, placebo-controlled, clinical trial of high-dose supplementation with vitamins C and E, beta carotene, and zinc for age-related macular degeneration and vision loss: AREDS report no. 8. Arch Ophthalmol 119: 1417-36

Aulchenko YS, Heutink P, Mackay I, Bertoli-Avella AM, Pullen J, Vaessen N, Rademaker TA, Sandkuijl LA, Cardon L, Oostra B, van Duijn CM (2004) Linkage disequilibrium in young genetically isolated Dutch population. Eur J Hum Genet 12: 527-34

Biino G, Palmas MA, Corona C, Prodi D, Fanciulli M, Sulis R, Serra A, Fossarello M, Pirastu M (2005) Ocular refraction: heritability and genome-wide search for eye morphometry traits in an isolated Sardinian population. Hum Genet 116: 152-9

Bild DE, Bluemke DA, Burke GL, Detrano R, Diez Roux AV, Folsom AR, Greenland P, Jacob DR, Jr., Kronmal R, Liu K, Nelson JC, O'Leary D, Saad MF, Shea S, Szklo M, Tracy RP (2002) Multi-ethnic study of atherosclerosis: objectives and design. Am J Epidemiol 156: 871-81

Clemons TE, Chew EY, Bressler SB, McBee W, Age-Related Eye Disease Study Research G (2003) National Eye Institute Visual Function Questionnaire in the Age-Related Eye Disease Study (AREDS): AREDS Report No. 10. Arch Ophthalmol 121: 211-7

Cornes BK, Khor CC, Nongpiur ME, Xu L, Tay WT, Zheng Y, Lavanya R, Li Y, Wu R, Sim X, Wang YX, Chen P, Teo YY, Chia KS, Seielstad M, Liu J, Hibberd ML, Cheng CY, Saw SM, Tai ES, Jonas JB, Vithana EN, Wong TY, Aung T (2012) Identification of four novel variants that influence central corneal thickness in multi-ethnic Asian populations. Hum Mol Genet 21: 437-45

Estrada K, Abuseiris A, Grosveld FG, Uitterlinden AG, Knoch TA, Rivadeneira F (2009) GRIMP: a web- and grid-based tool for high-speed analysis of large-scale genome-wide association using imputed data. Bioinformatics 25: 2750-2

Fan Q, Zhou X, Khor CC, Cheng CY, Goh LK, Sim X, Tay WT, Li YJ, Ong RT, Suo C, Cornes B, Ikram MK, Chia KS, Seielstad M, Liu J, Vithana E, Young TL, Tai ES, Wong TY, Aung T, Teo YY, Saw SM (2011) Genome-wide meta-analysis of five Asian cohorts identifies PDGFRA as a susceptibility locus for corneal astigmatism. PLoS Genet 7: e1002402

Foong AW, Saw SM, Loo JL, Shen S, Loon SC, Rosman M, Aung T, Tan DT, Tai ES, Wong TY (2007) Rationale and methodology for a population-based study of eye diseases in Malay people: The Singapore Malay eye study (SiMES). Ophthalmic Epidemiol 14: 25-35

Foran S, Wang JJ, Mitchell P (2003) Causes of visual impairment in two older population cross-sections: the Blue Mountains Eye Study. Ophthalmic Epidemiol 10: 215-25

Golding J, Pembrey M, Jones R, Team AS (2001) ALSPAC--the Avon Longitudinal Study of Parents and Children. I. Study methodology. Paediatr Perinat Epidemiol 15: 74-87

Harris TB, Launer LJ, Eiriksdottir G, Kjartansson O, Jonsson PV, Sigurdsson G, Thorgeirsson G, Aspelund T, Garcia ME, Cotch MF, Hoffman HJ, Gudnason V (2007) Age, Gene/Environment Susceptibility-Reykjavik Study: multidisciplinary applied phenomics. Am J Epidemiol 165: 1076-87

Hofman A, van Duijn CM, Franco OH, Ikram MA, Janssen HL, Klaver CC, Kuipers EJ, Nijsten TE, Stricker BH, Tiemeier H, Uitterlinden AG, Vernooij MW, Witteman JC (2011) The Rotterdam Study: 2012 objectives and design update. Eur J Epidemiol 26: 657-86

Holle R, Happich M, Lowel H, Wichmann HE, Group MKS (2005) KORA--a research platform for population based health research. Gesundheitswesen 67 Suppl 1: S19-25

Hughes K, Aw TC, Kuperan P, Choo M (1997) Central obesity, insulin resistance, syndrome X, lipoprotein(a), and cardiovascular risk in Indians, Malays, and Chinese in Singapore. J Epidemiol Community Health 51: 394-9

Khor CC, Ramdas WD, Vithana EN, Cornes BK, Sim X, Tay WT, Saw SM, Zheng Y, Lavanya R, Wu R, Wang JJ, Mitchell P, Uitterlinden AG, Rivadeneira F, Teo YY, Chia KS, Seielstad M, Hibberd M, Vingerling JR, Klaver CC, Jansonius NM, Tai ES, Wong TY, van Duijn CM, Aung T (2011) Genome-wide association studies in Asians confirm the involvement of ATOH7 and TGFBR3, and further identify CARD10 as a novel locus influencing optic disc area. Hum Mol Genet 20: 1864-72

Lavanya R, Jeganathan VS, Zheng Y, Raju P, Cheung N, Tai ES, Wang JJ, Lamoureux E, Mitchell P, Young TL, Cajucom-Uy H, Foster PJ, Aung T, Saw SM, Wong TY (2009) Methodology of the Singapore Indian Chinese Cohort (SICC) eye study: quantifying ethnic variations in the epidemiology of eye diseases in Asians. Ophthalmic Epidemiol 16: 325-36

Leibowitz HM, Krueger DE, Maunder LR, Milton RC, Kini MM, Kahn HA, Nickerson RJ, Pool J, Colton TL, Ganley JP, Loewenstein JI, Dawber TR (1980) The Framingham Eye Study monograph: An ophthalmological and epidemiological study of cataract, glaucoma, diabetic retinopathy, macular degeneration, and visual acuity in a general population of 2631 adults, 1973-1975. Surv Ophthalmol 24: 335-610

Li Y, Willer C, Sanna S, Abecasis G (2009) Genotype imputation. Annu Rev Genomics Hum Genet 10: 387-406

Li Y, Willer CJ, Ding J, Scheet P, Abecasis GR (2010) MaCH: using sequence and genotype data to estimate haplotypes and unobserved genotypes. Genet Epidemiol 34: 816-34

Li YJ, Goh L, Khor CC, Fan Q, Yu M, Han S, Sim X, Ong RT, Wong TY, Vithana EN, Yap E, Nakanishi H, Matsuda F, Ohno-Matsui K, Yoshimura N, Seielstad M, Tai ES, Young TL, Saw SM (2011) Genome-wide association studies reveal genetic variants in CTNND2 for high myopia in Singapore Chinese. Ophthalmology 118: 368-75

Mackey DA, Mackinnon JR, Brown SA, Kearns LS, Ruddle JB, Sanfilippo PG, Sun C, Hammond CJ, Young TL, Martin NG, Hewitt AW (2009) Twins eye study in Tasmania (TEST): rationale and methodology to recruit and examine twins. Twin Res Hum Genet 12: 441-54

Marchini J, Howie B, Myers S, McVean G, Donnelly P (2007) A new multipoint method for genome-wide association studies by imputation of genotypes. Nat Genet 39: 906-13

Medland SE, Zayats T, Glaser B, Nyholt DR, Gordon SD, Wright MJ, Montgomery GW, Campbell MJ, Henders AK, Timpson NJ, Peltonen L, Wolke D, Ring SM, Deloukas P, Martin NG, Smith GD, Evans DM (2010) A variant in LIN28B is associated with 2D:4D finger-length ratio, a putative retrospective biomarker of prenatal testosterone exposure. Am J Hum Genet 86: 519-25

Mitchell P, Smith W, Attebo K, Wang JJ (1995) Prevalence of age-related maculopathy in Australia. The Blue Mountains Eye Study. Ophthalmology 102: 1450-60

Nelis M, Esko T, Magi R, Zimprich F, Zimprich A, Toncheva D, Karachanak S, Piskackova T, Balascak I, Peltonen L, Jakkula E, Rehnstrom K, Lathrop M, Heath S, Galan P, Schreiber S, Meitinger T, Pfeufer A, Wichmann HE, Melegh B, Polgar N, Toniolo D, Gasparini P, D'Adamo P, Klovins J, Nikitina-Zake L, Kucinskas V, Kasnauskiene J, Lubinski J, Debniak T, Limborska S, Khrunin A, Estivill X, Rabionet R, Marsal S, Julia A, Antonarakis SE, Deutsch S, Borel C, Attar H, Gagnebin M, Macek M, Krawczak M, Remm M, Metspalu A (2009) Genetic structure of Europeans: a view from the North-East. PLoS One 4: e5472

Oexle K, Ried JS, Hicks AA, Tanaka T, Hayward C, Bruegel M, Gogele M, Lichtner P, Muller-Myhsok B, Doring A, Illig T, Schwienbacher C, Minelli C, Pichler I, Fiedler GM, Thiery J, Rudan I, Wright AF, Campbell H, Ferrucci L, Bandinelli S, Pramstaller PP, Wichmann HE, Gieger C, Winkelmann J, Meitinger T (2011) Novel association to the proprotein convertase PCSK7 gene locus revealed by analysing soluble transferrin receptor (sTfR) levels. Hum Mol Genet 20: 1042-7

Pardo LM, MacKay I, Oostra B, van Duijn CM, Aulchenko YS (2005) The effect of genetic drift in a young genetically isolated population. Ann Hum Genet 69: 288-95

Parssinen O, Jauhonen HM, Kauppinen M, Kaprio J, Koskenvuo M, Rantanen T (2010) Heritability of spherical equivalent: a population-based twin study among 63- to 76-year-old female twins. Ophthalmology 117: 1908-11

Rahi JS, Cumberland PM, Peckham CS (2011) Myopia over the lifecourse: prevalence and early life influences in the 1958 British birth cohort. Ophthalmology 118: 797-804

Raitakari OT, Juonala M, Ronnemaa T, Keltikangas-Jarvinen L, Rasanen L, Pietikainen M, Hutri-Kahonen N, Taittonen L, Jokinen E, Marniemi J, Jula A, Telama R, Kahonen M, Lehtimaki T, Akerblom HK, Viikari JS (2008) Cohort profile: the cardiovascular risk in Young Finns Study. Int J Epidemiol 37: 1220-6

Saw SM, Shankar A, Tan SB, Taylor H, Tan DT, Stone RA, Wong TY (2006) A cohort study of incident myopia in Singaporean children. Invest Ophthalmol Vis Sci 47: 1839-44

Sim X, Ong RT, Suo C, Tay WT, Liu J, Ng DP, Boehnke M, Chia KS, Wong TY, Seielstad M, Teo YY, Tai ES (2011) Transferability of type 2 diabetes implicated loci in multi-ethnic cohorts from Southeast Asia. PLoS Genet 7: e1001363

Smith EN, Chen W, Kahonen M, Kettunen J, Lehtimaki T, Peltonen L, Raitakari OT, Salem RM, Schork NJ, Shaw M, Srinivasan SR, Topol EJ, Viikari JS, Berenson GS, Murray SS (2010) Longitudinal genome-wide association of cardiovascular disease risk factors in the Bogalusa heart study. PLoS Genet 6

Spector TD, Williams FM (2006) The UK Adult Twin Registry (TwinsUK). Twin Res Hum Genet 9: 899-906

Steffens M, Lamina C, Illig T, Bettecken T, Vogler R, Entz P, Suk EK, Toliat MR, Klopp N, Caliebe A, Konig IR, Kohler K, Ludemann J, Diaz Lacava A, Fimmers R, Lichtner P, Ziegler A, Wolf A, Krawczak M, Nurnberg P, Hampe J, Schreiber S, Meitinger T, Wichmann HE, Roeder K, Wienker TF, Baur MP (2006) SNP-based analysis of genetic substructure in the German population. Hum Hered 62: 20-9

Tonjes A, Koriath M, Schleinitz D, Dietrich K, Bottcher Y, Rayner NW, Almgren P, Enigk B, Richter O, Rohm S, Fischer-Rosinsky A, Pfeiffer A, Hoffmann K, Krohn K, Aust G, Spranger J, Groop L, Bluher M, Kovacs P, Stumvoll M (2009) Genetic variation in GPR133 is associated with height: genome wide association study in the self-contained population of Sorbs. Hum Mol Genet 18: 4662-8

Vitart V, Bencic G, Hayward C, Herman JS, Huffman J, Campbell S, Bucan K, Zgaga L, Kolcic I, Polasek O, Campbell H, Wright A, Vatavuk Z, Rudan I (2010a) Heritabilities of ocular biometrical traits in two croatian isolates with extended pedigrees. Invest Ophthalmol Vis Sci 51: 737-43

Vitart V, Bencic G, Hayward C, Skunca Herman J, Huffman J, Campbell S, Bucan K, Navarro P, Gunjaca G, Marin J, Zgaga L, Kolcic I, Polasek O, Kirin M, Hastie ND, Wilson JF, Rudan I, Campbell H, Vatavuk Z, Fleck B, Wright A (2010b) New loci associated with central cornea thickness include COL5A1, AKAP13 and AVGR8. Hum Mol Genet 19: 4304-11

Vithana EN, Aung T, Khor CC, Cornes BK, Tay WT, Sim X, Lavanya R, Wu R, Zheng Y, Hibberd ML, Chia KS, Seielstad M, Goh LK, Saw SM, Tai ES, Wong TY (2011) Collagen-related genes influence the glaucoma risk factor, central corneal thickness. Hum Mol Genet 20: 649-58

Wichmann HE, Gieger C, Illig T, Group MKS (2005) KORA-gen--resource for population genetics, controls and a broad spectrum of disease phenotypes. Gesundheitswesen 67 Suppl 1: S26-30

Zeller T, Wild P, Szymczak S, Rotival M, Schillert A, Castagne R, Maouche S, Germain M, Lackner K, Rossmann H, Eleftheriadis M, Sinning CR, Schnabel RB, Lubos E, Mennerich D, Rust W, Perret C, Proust C, Nicaud V, Loscalzo J, Hubner N, Tregouet D, Munzel T, Ziegler A, Tiret L, Blankenberg S, Cambien F (2010) Genetics and beyond--the transcriptome of human monocytes and disease susceptibility. PLoS One 5: e10693
